# Functional inhibition of acid sphingomyelinase by Fluphenazine triggers hypoxia-specific tumor cell death

**DOI:** 10.1038/cddis.2017.130

**Published:** 2017-03-30

**Authors:** Saskia Klutzny, Ralf Lesche, Matthias Keck, Stefan Kaulfuss, Andreas Schlicker, Sven Christian, Carolyn Sperl, Roland Neuhaus, Jeffrey Mowat, Michael Steckel, Björn Riefke, Stefan Prechtl, Karsten Parczyk, Patrick Steigemann

**Affiliations:** 1 Drug Discovery, Bayer AG, Berlin 13353, Germany; 2 Department of Bioanalytics, Institute for Biotechnology, Technical University of Berlin, Berlin, Germany

## Abstract

Owing to lagging or insufficient neo-angiogenesis, hypoxia is a feature of most solid tumors. Hypoxic tumor regions contribute to resistance against antiproliferative chemotherapeutics, radiotherapy and immunotherapy. Targeting cells in hypoxic tumor areas is therefore an important strategy for cancer treatment. Most approaches for targeting hypoxic cells focus on the inhibition of hypoxia adaption pathways but only a limited number of compounds with the potential to specifically target hypoxic tumor regions have been identified. By using tumor spheroids in hypoxic conditions as screening system, we identified a set of compounds, including the phenothiazine antipsychotic Fluphenazine, as hits with novel mode of action. Fluphenazine functionally inhibits acid sphingomyelinase and causes cellular sphingomyelin accumulation, which induces cancer cell death specifically in hypoxic tumor spheroids. Moreover, we found that functional inhibition of acid sphingomyelinase leads to overactivation of hypoxia stress-response pathways and that hypoxia-specific cell death is mediated by the stress-responsive transcription factor ATF4. Taken together, the here presented data suggest a novel, yet unexplored mechanism in which induction of sphingolipid stress leads to the overactivation of hypoxia stress-response pathways and thereby promotes their pro-apoptotic tumor-suppressor functions to specifically kill cells in hypoxic tumor areas.

Rapid cell growth and high metabolic rates of cancer cells in combination with insufficient or disorganized neovascularization can lead to the development of hypoxic or anoxic tumor regions.^1, 2, 3^ Intratumoral hypoxia is a common feature of most solid tumors^2, 4, 5^ and inversely correlates with clinical efficacy of cancer therapies and clinical outcome.^5, 6, 7, 8, 9, 10, 11^ Therefore, targeting cancer cells in hypoxic tumor areas is an important strategy for cancer treatment.

One of the main strategies for targeting hypoxic tumor cells is to inhibit or modulate hypoxia-survival pathways. Under hypoxia, cancer cells activate different but partially overlapping stress-response pathways to adapt cellular metabolism and promote pro-survival pathways.^9^ These include the stabilization of hypoxia-inducible transcription factors (HIF), the activation of endoplasmic reticulum (ER) stress pathways, as well as the inhibition of mTOR signaling.^12^

Although hypoxic signaling via the known pathways is relatively well characterized,^9, 13^ less is known how hypoxia-response pathways interact to orchestrate different hypoxic responses and integrate other stress signals, for example, to balance pro-survival and pro-apoptotic signals.^14, 15^ Furthermore, apart from the canonical hypoxia pathways, little is known about the adaptive mechanisms needed for cancer cells to survive severe hypoxia. Accordingly, so far there are only few drugs that act to specifically target hypoxic or anoxic cells in tumors.^8, 16^

Therefore, we established a screening-compatible HCT116 tumor spheroid model that mimics regions of severe hypoxia in tumors and performed a phenotypic screen on a library of known bioactive small molecules for the identification of hypoxia-sensitizing compounds that specifically induce cell death in hypoxic or anoxic tumor spheroids.

By this, we identified and validated GLUT or glycolysis inhibition as potential strategy to specifically kill hypoxic cells. Additionally, four highly hypoxia-selective compounds with novel mode of action were identified that specifically induce cell death in hypoxic spheroids and show no effects in spheroids cultured under normoxia. Of these substances, Fluphenazine, an antipsychotic phenothiazine drug, could be validated as novel hypoxia-selective cell death inducer. Fluphenazine induces lysosomal stress, functionally inhibits the lysosomal enzyme acid sphingomyelinase (ASMase) and leads to the accumulation of cellular sphingomyelin (SM). Moreover, SM supplementation phenocopies the effects of Fluphenazine in hypoxic spheroids. Importantly, Fluphenazine acts differently than known lysosomal-disrupting agents^17, 18^ or inhibitors of lysosomal acidification.^19^ Furthermore, by deep sequencing we show that Fluphenazine induces overactivity of hypoxia stress-response pathways and causes hypoxia-specific cell death via the stress-response transcription factor ATF4.

## Results

### Spheroids as *in vitro* model for tumor hypoxia

**Figure 1:**
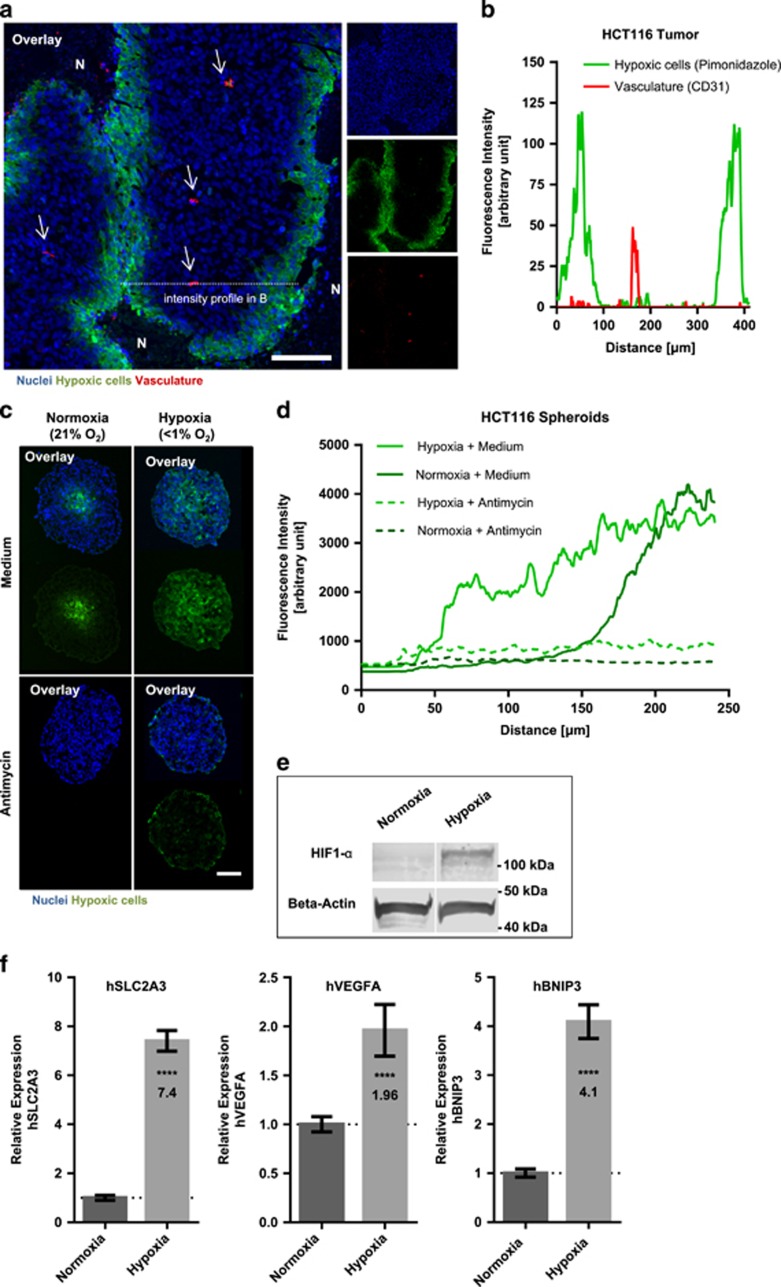
HCT116 tumor spheroids incubated in hypoxia mimic hypoxic tumor regions distal from blood vessels in HCT116 xenografts. (**a**) IHC staining of HCT116 colon cancer xenograft tumor sections. HCT116 tumor cryosections were stained for the exogenous hypoxia marker pimonidazole (Hypoxyprobe, green), CD31 as marker for blood vessel (red) and the nuclear marker Hoechst (blue). Arrows indicate blood vessels. N=necrotic region. Dashed line showing region for measuring intensity profile in panel (**b**). Scale bar 100 *μ*m. (**b**) Line scan (dashed line in panel (**a**)) through HCT116 tumor section showing intensity profile of pimonidazole and CD31 staining. (**c**) Cryosections of HCT116 spheroids. Spheroids were treated for 3 days in normoxia or hypoxia with or without the complex III inhibitor Antimycin (200 nM). Nuclei were stained by Hoechst (blue) and hypoxic areas with anti-pimonidazole (green). Scale bar 100 *μ*m. (**d**) Intensity profile pimonidazole staining (average of multiple spheroids, *n*=5–10) from spheroid border to spheroid core region of HCT116 spheroids. (**e**) Western blotting analysis of HCT116 spheroids incubated for 24 h in normoxia or hypoxia. Beta-actin was used as an internal control. Representative data of multiple experiments shown (*n*=3). (**f**) Real-time quantitative PCR gene expression analysis of HIF target genes in HCT116 spheroids incubated for 24 h in normoxia or hypoxia. Ct values of each sample were normalized with the internal control RPL32 and normalized to the normoxia sample. Bars show mean with S.D. (*n*=3). **** = *P*-value smaller 0.0001

Fast growth of cancer cells and lagging neo-angiogenesis can lead to hypoxia in a large portion of the tumor, especially in cells located distal to supplying blood vessels. Figures 1a and b show the distribution of hypoxic areas in tumor xenograft sections of HCT116 colon cancer cells with hypoxic and dormant regions increasing with growing distance from supplying blood vessels (see also^1, 9^).

Hypoxic and nutrient-depleted conditions found in tumor tissue can be mimicked in a 3D cell culture setting and tumor spheroids are being increasingly used to better reflect physiological conditions of tumor cell growth *in vitro*. To identify compounds that specifically target cells in hypoxic or anoxic tumor regions, we aimed to establish conditions of severe hypoxia in HCT116 colon cancer tumor spheroids. Cryosections from HCT116 tumor spheroids cultured under normoxic conditions show pimonidazole staining in the core region of the spheroid. However, this region expands to the outer spheroid cell layer in spheroids that were cultured in hypoxic conditions (see Figure 1c and hypoxia profile in Figure 1d, see Material and Methods section for spheroid formation and incubation).

In tumor tissue, hypoxic gradients develop by cellular respiration of intervening cells and promote the development of hypoxic regions in cells distal from oxygen-supplying blood vessels.^1^ Accordingly, pimonidazole staining of hypoxic regions in HCT116 tumor spheroids is diminished after addition of an inhibitor of cellular respiration (Figures 1c and d), indicating that hypoxic gradients in spheroids are also established by cellular oxygen consumption.

Additionally supporting the use of spheroids cultured under hypoxia as model system for hypoxic tumor regions, HIF-1-*α* shows strong accumulation in tumor spheroids cultured under hypoxia while it is only faintly detected in normoxic conditions (Figure 1e). Moreover, mRNA expression of HIF-1-*α* target genes are upregulated in tumor spheroids cultured under hypoxia (Figure 1f).

Taken together, these data show that HCT116 tumor spheroids cultured in reduced oxygen conditions are strongly hypoxic and activate hypoxia adaption pathways. Thus they represent a suitable model system for the identification of compounds that target tumor hypoxia or anoxia.

### Screen for the identification of compounds that induce hypoxia-specific cell death

We hypothesized that targeting hypoxia adaption pathways necessary for cellular survival should induce cell death in HCT116 tumor spheroids incubated under hypoxia, while showing less or no effect in spheroids cultured under normoxia. In order to identify such compounds, we established HCT116 tumor spheroid formation in screening-compatible 384-well microtiter plates (see Materials and Methods section) and screened a drug library of known bioactive substances on HCT116 colon cancer spheroids cultured under hypoxia or normoxia (*n*=4 per compound).

From 468 tested compounds, 43 induced a significant increase in staining for cell death under hypoxia (at least 50% intensity of dead cell staining normalized to controls). For 16 of these compounds, cell death staining was at least two times stronger under hypoxia compared with normoxia (for a complete list of all tested compounds, see Supplementary Table S1). Nine of the 16 hit compounds could be validated as hypoxia specific, showing concentration-dependent cell death induction under hypoxia and no effects or effects only at significantly higher concentrations under normoxia (see Table 1 and Supplementary Figure S2). To exclude cell-line-specific effects, all confirmed hits were also profiled in T47D breast cancer tumor spheroids and showed similar effects (Supplementary Table S2).

Taken together, from 468 compounds of a known bioactives library, 9 compounds could be identified as hypoxia-sensitizing compounds, showing an increase in cell death staining specifically when incubated with tumor spheroids under hypoxia.

### GLUT or glycolysis inhibition induces hypoxia-specific cell death

A major characteristic of cellular hypoxia is the repression of the respiratory chain, which forces cells to switch from cellular respiration to glycolysis for energy production. Therefore, we speculated that hypoxia-specific hits could act by sensitizing cells to hypoxia by interfering with glycolysis. To identify such compounds, we tested the effects of all hits under normoxia in co-incubation with a respiratory chain inhibitor (complex III inhibitor Antimycin). Respiratory chain inhibition renders cells dependent on glycolysis for energy production and survival and co-incubation with a glucose transport (GLUT) or glycolysis inhibitor leads to synthetic lethality also under normoxia.^20^

Two compounds from the hit list, Cytochalasin B and E6 Berbamine, showed synthetic lethality on HCT116 spheroids cultured under normoxia when incubated with Antimycin (see Table 1 and Supplementary Figure S2). Indeed, Cytochalasin B is a long-known GLUT inhibitor.^21^ In order to verify glycolysis as an important factor for cellular survival under hypoxia, we also tested a glycolysis inhibitor (2-deoxy-D-glucose (2-DG)) and incubation in medium with low glucose. In both cases, we found similar hypoxia-specific effects in HCT116 tumor spheroids and synthetic lethality after co-incubation with Antimycin under normoxia (Supplementary Figure S3).

Taken together, these data indicate that limiting glucose supply or inhibition of glycolysis could be an option to kill cells in hypoxic tumor areas. A detailed analysis of GLUT inhibitors for cancer therapy is covered elsewhere,^22, 23, 24^ and we concentrated our studies on the remaining hits with a novel mode of action.

### Hypoxia-sensitizing compounds alter cellular lipid composition by inhibiting ASMase

Seven of the identified hits displayed a phenotype different from GLUT or glycolysis inhibitors, showing no synthetic lethality in tumor spheroids when co-incubated with Antimycin under normoxia (see Supplementary Figure S2). However, three of these substances induced cell death or proliferation inhibition also in hypoxic or normoxic 2D culture conditions and therefore were not further evaluated (see Table 1). Strikingly, co-incubation with the respiratory chain inhibitor Antimycin, which prevents the establishment of hypoxic gradients in spheroids (see Figure 1c), almost completely prevented induction of cell death by the remaining four hit compounds in tumor spheroids cultured under hypoxia (Table 1 and Supplementary Figure S2).

**Figure 2:**
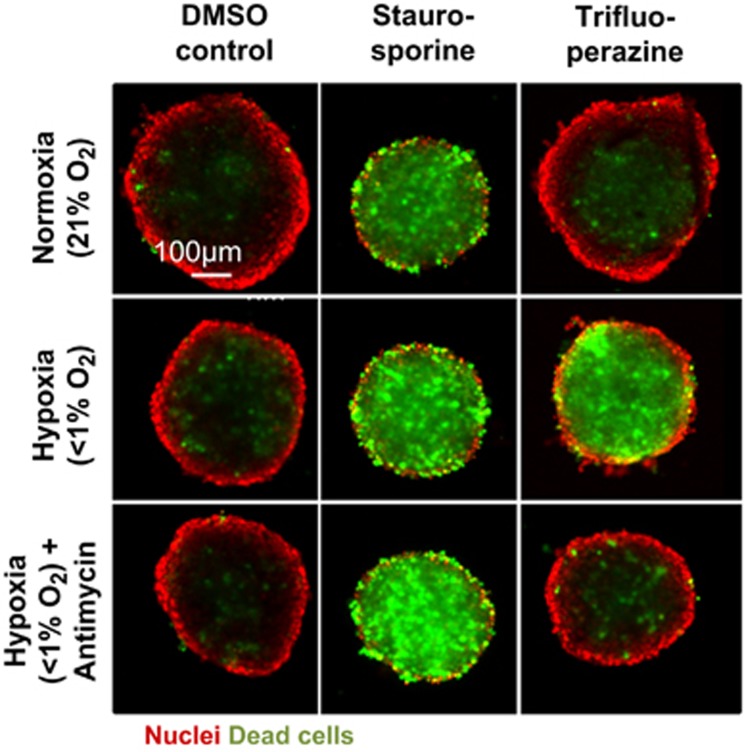
High-content screen on HCT116 spheroids identifies hypoxia-sensitizing compounds. HCT116 spheroids were grown for 4 days in normoxic conditions, followed by 3 days of compound treatment (DMSO control, Staurosporine 10 *μ*M or Trifluoperazine 5 *μ*M) in normoxia, hypoxia or hypoxia+Antimycin 200 nM. Spheroid nuclei were stained with Hoechst (red) and dead cells were stained with SytoxGreen (green). Hypoxia-sensitizing compound Trifluoperazine specifically induces cell death in spheroids cultured under hypoxia. Representative images of multiple experiments shown (*n*≥3). Scale bar 100 *μ*m

The most potent compound of this novel class of hypoxia-selective hits is Trifluoperazine (see Figure 2 and Table 1), an antipsychotic of the phenothiazine class, which is known to inhibit dopamine receptors.^25^ However, while other phenothiazines such as Fluphenazine, Chlorpromazine or Thioridazine showed similar hypoxia-specific effects (see Table 1 and Supplementary Figure S2), other structurally unrelated antipsychotics with activity against dopamine receptors, such as Haloperidol, Amisulpride, Pimozide or Fluspirilene,^26, 27, 28, 29^ showed no hypoxia-specific effects (data not shown). Therefore, we speculated that these compounds specifically kill cells in hypoxic tumor spheroid regions independently of their known biological function. For further evaluation of hypoxia-sensitizing compounds, we concentrated on Fluphenazine as one of the most potent compounds with high hypoxia specificity in spheroids (1.63 *μ*M under hypoxia, >10 *μ*M under normoxia) (Table 1).

Based on the compound structure, many of the identified hits, including Fluphenazine, can be characterized as ‘cationic amphiphilic' drugs (CAD) (see Supplementary Table S3).^30^ CADs can accumulate in lysosomes, cause lysosome dysfunction and lysosome membrane permeabilization (LMP).^17, 31, 32^ Accordingly, most identified hypoxia-sensitizing compounds have been reported as lysosomotropic substances.^33, 34, 35, 36, 37^

**Figure 3:**
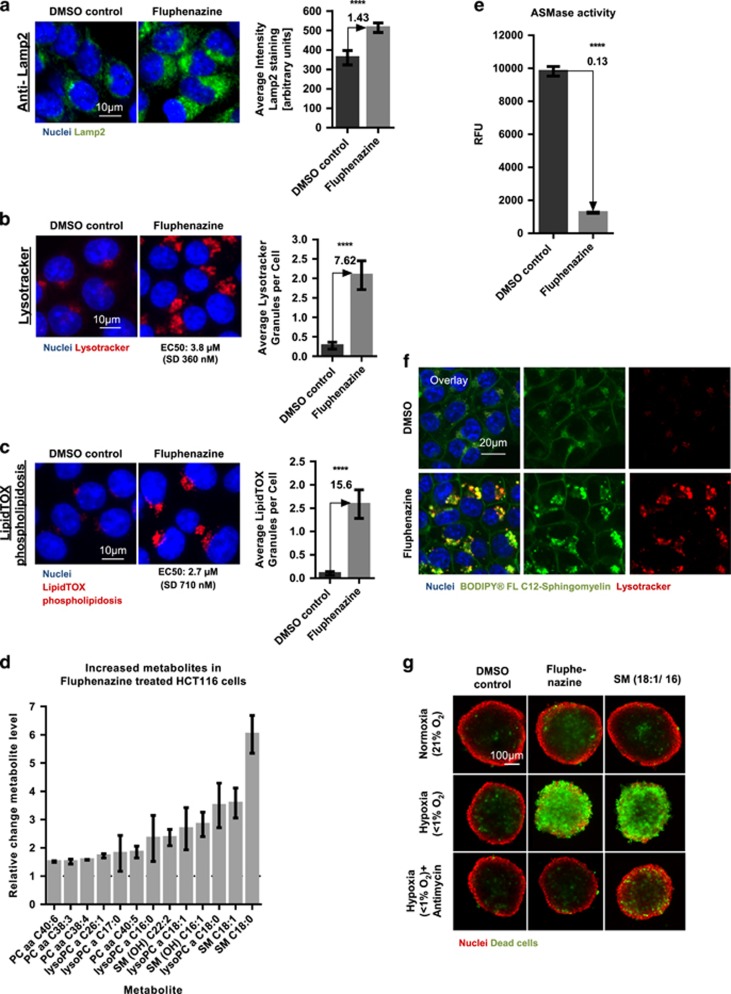
Fluphenazine induces lysosomal stress and inhibits ASMase. (**a**–**c**) HCT116 cells were treated for 24 h with either DMSO control or 5 *μ*M Fluphenazine. Cells were either stained for (**a**) lysosome marker Lamp2 (**b**) for acidic vesicles using Lysotracker or (**c**) for the accumulation of undigested phospholipids using the LipidTOX phospholipidosis staining. Nuclei were stained with Hoechst. Quantification of stainings shown on right hand side. Bars show mean with S.D. (*n*=3). **** = *P*-value smaller 0.0001. Scale bar 10 *μ*m. (**d**) Metabolomics analysis of 188 endogenous metabolites identifies the accumulation of SMs in Fluphenazine-treated cells. For full profile, please see Supplementary Figure S4. HCT116 cells treated for 24 h with 5 *μ*M Fluphenazine (compared with DMSO control). Bars show mean with S.D. (*n*=2, 4 replicates per experiment). **** = *P*-value smaller 0.0001. lysoPC: lysophosphatidylcholine. (**e**) ASMase activity in HCT116 cells treated for 24 h with either DMSO control or 5 *μ*M Fluphenazine shows strong reduction after Fluphenazine treatment. Bars show mean with S.D. (*n*=3). **** = *P*-value smaller 0.0001. (**f**) HCT116 cells were co-incubated overnight with either DMSO control+1 *μ*M BODIPY FL C12-Sphingomyelin or 5 *μ*M Fluphenazine+1 *μ*M BODIPY FL C12-Sphingomyelin. Nuclei were stained with Hoechst and Lysosomes with Lysotracker. Scale bar 20 *μ*m. (**g**) HCT116 spheroids were grown for 4 days under normoxic conditions, followed by 3 days of compound treatment (DMSO control, 5 *μ*M Fluphenazine, 100 *μ*M *N*-Palmitoyl-D-Sphingomyelin (18:1/16)) and incubation either in normoxia, hypoxia or hypoxia and 200 nM Antimycin. Spheroid nuclei were stained with Hoechst (red) and dead cells were stained with SytoxGreen (green). Representative images of multiple experiments are shown (*n*≥3). Scale bar 100 *μ*m

Therefore, we first investigated the impact of Fluphenazine treatment on lysosomes. By Lamp2 antibody staining, a lysosomal-associated protein, we found that Fluphenazine leads to an aggregation of lysosomes (Figure 3a). Accordingly, Fluphenazine treatment results in a dose-dependent accumulation of acidic vesicles seen by Lysotracker staining at an EC50 of 3.8 *μ*M (S.D. 360 nM), (see Figure 3b).

Lysosomes are a major site of cellular phospholipid metabolism. Lysosomal stress or damage induced by CADs can induce phospholipidosis, the accumulation of phospholipids in cells and tissues.^30, 38^ To test for lysosome functionality, we therefore measured the accumulation of lipids using the LipidTOX Phospholipidosis Detection Kit. Fluphenazine led to a dose-dependent accumulation (EC50 of 2.74 *μ*M, S.D. 710 nM) of fluorescent phospholipids (see Figure 3c). Similar results were obtained for Trifluoperazine, Tamoxifen and ML9 (data not shown).

Taken together, these data indicate that Fluphenazine impairs lysosomal functionality at concentrations required for induction of cell death in hypoxic spheroids (compare with Table 1).

To further investigate this, we profiled the metabolic signature of 188 metabolites, including different lipids of Fluphenazine-treated cells by mass spectrometry (see Supplementary Figure S4). Fluphenazine treatment led to a strong accumulation of SMs, phosphocholines (PCs) and lysophosphatidylcholines (Figure 3d). SMs are a major component of cellular lipid membranes. In lysosomes, they are converted to ceramide and PC by the lysosomal enzyme ASMase. Therefore, we speculated that Fluphenazine could interfere with ASMase function. Indeed, cell extracts of HCT116 cells treated for 24 h with Fluphenazine before harvesting showed a reduction of ASMase activity (Figure 3e). However, when cell extracts from untreated cells or recombinant ASMase were treated with Fluphenazine, we found no reduction in ASMase activity (data not shown). Therefore, we conclude that Fluphenazine functionally inhibits ASMase and leads to alterations in cellular sphingolipid composition. Accordingly, exogenously added fluorescent SM shows strong lysosomal accumulation after Fluphenazine treatment (Figure 3f).

To test whether Fluphenazine-induced hypoxia-specific cell death in spheroids could be a consequence of cellular SM accumulation, tumor spheroids were incubated with different concentrations of exogenously added SM. Indeed, exogenous addition of *N*-Palmitoyl-D-Sphingomyelin (18:1/16) led to a dose-dependent induction of cell death in hypoxic (58.4 *μ*M, S.D. 1.9 *μ*M) but not in normoxic spheroids. Additionally, similar to Fluphenazine treatment, co-treatment of SM with a respiratory chain inhibitor led to a rescue of cell death induction in tumor spheroids (Figure 3g). In contrast, the addition of the direct SM metabolites ceramide or PC showed no effect on cell viability nor did it prevent Fluphenazine-induced cell death (data not shown). Taken together, we conclude that hypoxia-specific induction of cell death in spheroids is caused by cellular SM accumulation and not by the accumulation of SM metabolites.

### Fluphenazine functionally inhibits ASMase distinct from known lysosomotropic substances

**Figure 4:**
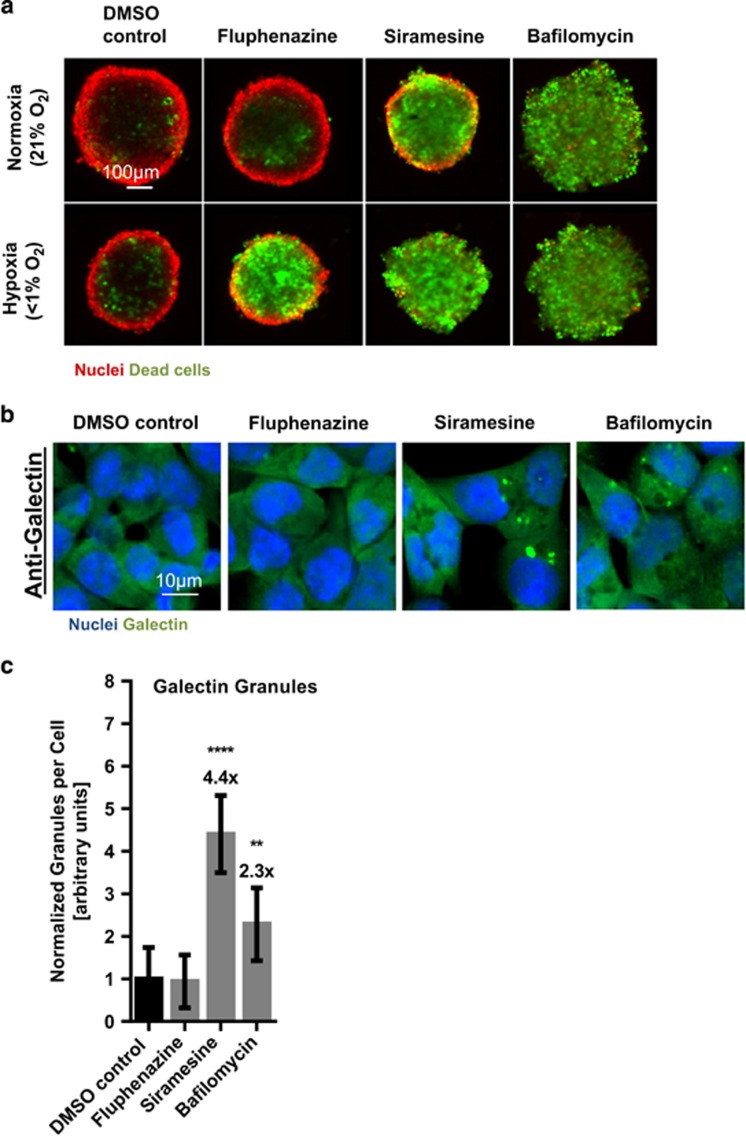
Fluphenazine induces lysosomal stress distinct from known lysosomotropic substances. (**a**) HCT116 spheroids were grown for 4 days in normoxic conditions, followed by 3 days of compound treatment (DMSO control, Fluphenazine 5 *μ*M, Siramesine 5 *μ*M or Bafilomycin 200 nM) and incubation either in normoxia or hypoxia. Spheroid nuclei were stained with Hoechst (red) and dead cells were stained with SytoxGreen (green). Only Fluphenazine induces hypoxia-specific cell death. Representative images of multiple experiments shown (*n*≥3). Scale bar 100 *μ*m. (**b**) HCT116 cells were treated for 24 h with DMSO control, 5 *μ*M Fluphenazine, 5 *μ*M Siramesine or 200 nM Bafilomycin. Cells were stained for Galectin (*n*=4). Scale bar 10 *μ*M. (**c**) Quantification of galectin puncta formation from panel (**b**). Bars show mean with S.D. ** = *P*-value between 0.001 and 0.01, **** = P-value smaller 0.0001

Interestingly, LMP compounds such as Siramesine have been shown to inhibit ASMase and have been proposed as anticancer drugs for the treatment of multidrug-resistant cancers.^17, 18^ By interfering with lysosomal membrane integrity and ASMase membrane localization, they are thought to act as functional inhibitors of ASMase.^32^ However, as lysosomes are important organelles that cover a wide range of cellular functions, disrupting lysosomes in general will impair a variety of cellular functions and has been proposed to be generally cytotoxic to most cells.^39^ Indeed, we find that the LMP-inducing compound Siramesine, as well as the V-ATPase inhibitor Bafilomycin A1, which also generally disrupts lysosomal functions by preventing lysosomal acidification,^19^ do not act as hypoxia-sensitizing compounds as they induce cell death in spheroids or in 2D cell culture under hypoxia or normoxia alike (see Table 2 and Figure 4a). This strongly contrasts with the ASMase inhibitor Fluphenazine, which is inactive in spheroids under normoxia and in 2D culture conditions (see Table 1). Moreover, while Siramesine and Bafilomycin lead to the formation of galectin puncta at lysosomes, which is an indicator for LMP,^40^ this could not be detected in Fluphenazine-treated cells (Figures 4b and c). Therefore, we conclude that Fluphenazine targets lysosomal functions without destabilizing lysosomal membranes.

### Fluphenazine induces HIF overactivation in conditions of high HIF background levels

We speculated that hypoxia-sensitizing compounds such as Fluphenazine could induce hypoxia-specific cell death in tumor spheroids by interfering with hypoxia adaption pathways. The transcription factor family of HIFs is selectively stabilized under hypoxia and regulates the transcription of several genes important for a cellular response to low oxygen levels.

**Figure 5:**
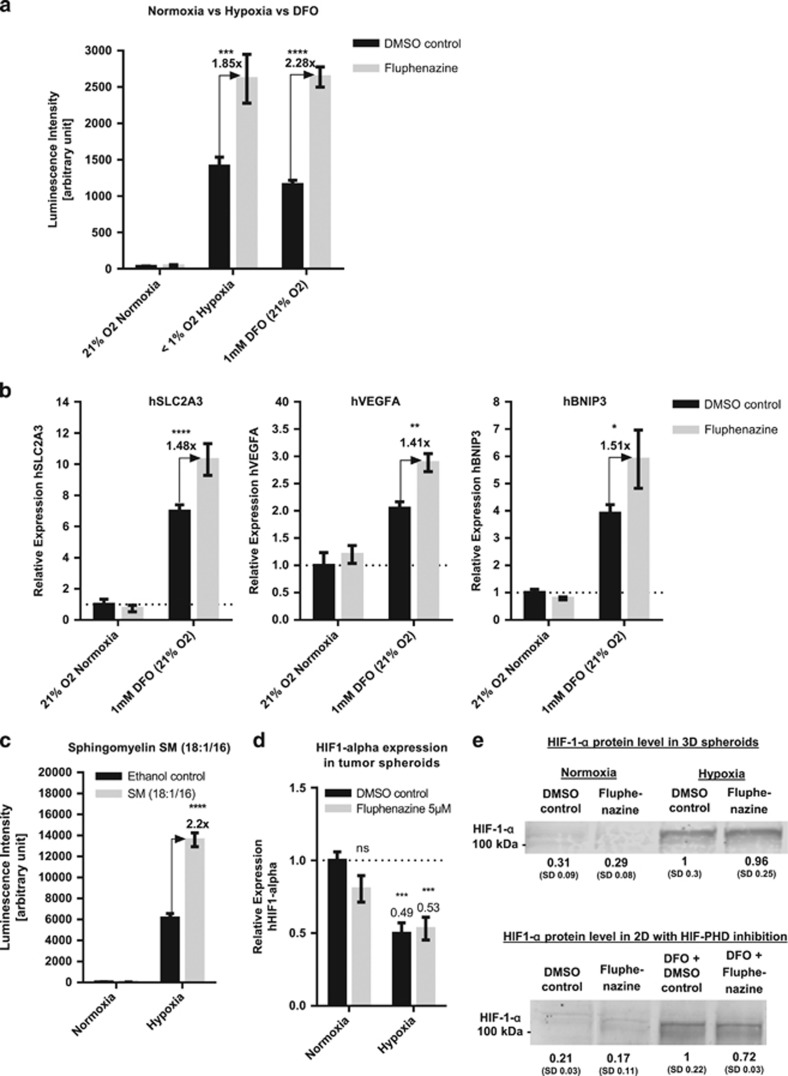
Fluphenazine induces HIF overactivation in conditions of high HIF background levels. (**a**) HIF activity reporter cells HCT116-HRE-Luc were treated either with DMSO control or 10 *μ*M Fluphenazine and incubated for 16 h in normoxia, hypoxia or in normoxia with the HIF-PH inhibitor DFO. After incubation, cells were lysed and luciferase activity was measured. Bars show mean with S.D. (*n*=3). (**b**) HCT116 cells were treated for 24 h with or without DFO (1 mM) and additionally with either DMSO control or Fluphenazine (5 *μ*M). Gene expression analysis was performed for three HIF1 target genes (SLC2A3, VEGFA and BNIP3) by real-time quantitative PCR (RT-qPCR). hRP-L32 was used as reference gene and relative expression level were normalized to the untreated control (no DFO, DMSO). Bars show mean with S.D. (*n*=3). (**c**) HCT116-HRE-Luc cells were treated with either Ethanol control or 100 *μ*M SM (18:1/16) and incubated for 24 h either in normoxia or hypoxia. After incubation, cells were lysed and luciferase activity was measured (*n*=3). (**d**) RT-qPCR analysis of HIF-1-a mRNA level of HCT116 Spheroids treated for 24 h in normoxia or hypoxia with either DMSO control or 5 *μ*M Fluphenazine. hRP-L32 was used as reference gene and the relative expression level were normalized with the untreated control (normoxia DMSO control). Bars show mean with S.D. (*n*=3). (**e**) Western blotting analysis of HIF-1-a protein expression in HCT116 spheroids or HCT116 cells grown in 2D that were treated for 24 h with either DMSO control or 5 *μ*M Fluphenazine under normoxia, hypoxia or normoxia+1 mM DFO. Representative data of multiple experiments are shown (*n*=3). Intensity values were normalized to loading control and DMSO-treated controls under hypoxia (upper row) or DMSO controls co-incubated with DFO (lower row). Beta-actin was used as an internal control (not shown). * = *P*-value between 0.01 and 0.05, ** = *P*-value between 0.001 and 0.01, *** = *P*-value between 0.0001 and 0.001, **** = *P*-value smaller 0.0001

We therefore asked whether hypoxia-sensitizing compounds interfere with HIF signaling. For this, a HCT116 reporter cell line that expresses luciferase under the control of a hypoxia-response element (HRE)^41^ was used to measure HIF transcriptional activity (HRE-Luc assay; see Material and Methods section). Although the reporter shows no activity under normoxia, the signal increases significantly under hypoxia or after co-incubation with the hypoxia-mimetic Deferoxamine mesylate (DFO)^42^ (see Figure 5a). Interestingly, Fluphenazine has no effect on the luminescence signal under normoxia but shows an approximately two-fold stronger signal compared with controls in conditions of high HIF background activity (hypoxia or DFO treatment; see Figure 5a and Supplementary Figure S5).

To further evaluate this, we measured the effect of Fluphenazine on endogenous HIF target gene expression by real-time quantitative PCR. Pro-survival (*SLC2A3* (GLUT3) and *VEGFA*) as well as pro-apoptotic HIF target genes (*BNIP3*) were upregulated in HIF-stabilized conditions (DFO treatment), while all measured mRNA levels showed no significant expression level changes under normoxic growth conditions upon Fluphenazine treatment (Figure 5b). Furthermore, similar to Fluphenazine treatment exogenous SM supplementation led to a 2.2-fold stronger luminescence signal compared with controls in hypoxic conditions (Figure 5c). Therefore, we conclude that Fluphenazine induces HIF transcriptional overactivity in conditions of high HIF basal levels possibly by inducing cellular SM accumulation.

HIF is regulated at different levels, including translational and transcriptional control, as well as protein stability and activity.^16, 43^ Fluphenazine treatment did not lead to alterations in HIF-1-*α* transcript levels (Figure 5d) and no differences in HIF-1-*α* protein levels could be observed (Figure 5e). Hence, we conclude that Fluphenazine leads to an activation of HIF-1-dependent transcription, downstream of HIF-1-*α* protein stabilization or accumulation, for example, on the level of activity regulation by posttranslational protein modification or interaction with transcriptional co-factors, such as ATF4.^44^

### Fluphenazine induces stress-response pathways

To obtain a broader picture of transcriptional regulation induced by Fluphenazine, we determined the transcript level of all protein-encoding genes (~20 000 genes) by deep sequencing of HCT116 cells treated with either DFO or DFO together with Fluphenazine.

**Figure 6:**
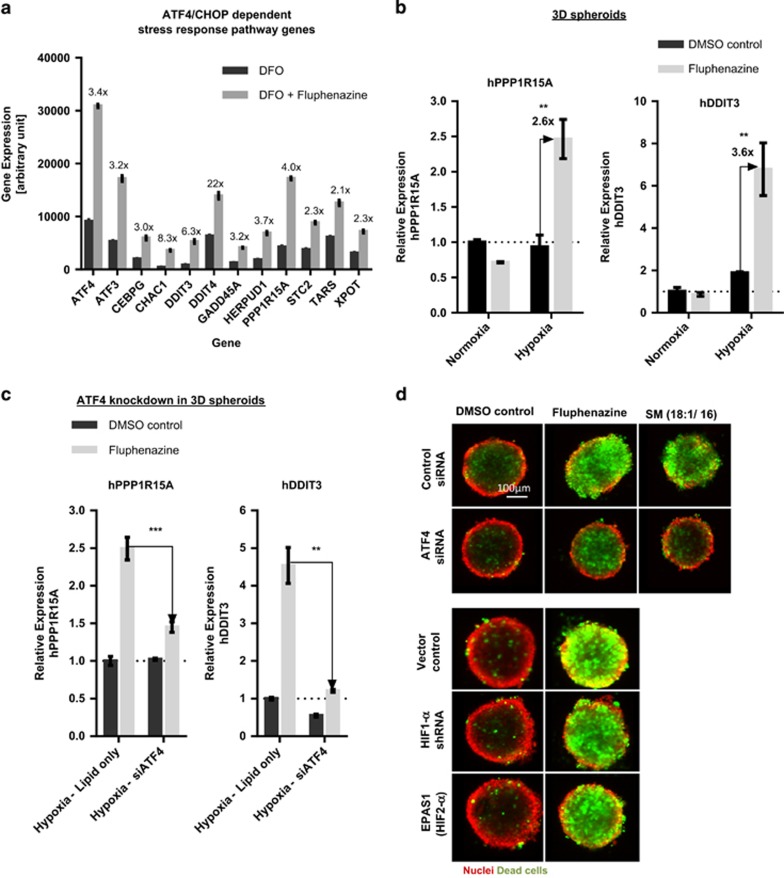
Fluphenazine induces ATF4 overactivation in hypoxic spheroids. (**a**) Analysis of all protein-encoding genes by deep sequencing of HCT116 cells treated for 24 h with either 1 mM DFO or 1 mM DFO+5 *μ*M Fluphenazine (see also Supplementary Figure S6) showed upregulation of ATF4/CHOP-dependent stress-response pathway upon Fluphenazine and DFO co-treatment. Bars show mean with S.D. (*n*=1, median of 4 replicates). (**b**) Gene expression analysis (RT-qPCR) of ATF4 pro-apoptotic target genes in HCT116 spheroids treated for 24 h in normoxia or hypoxia with DMSO control or 5 *μ*M Fluphenazine. Ct values of each sample were normalized with the internal control RPL32 and normalized to the normoxia DMSO control. Bars show mean with S.D. (*n*=3). (**c**) HCT116 cells were incubated with ATF4 siRNA or lipid only control and grown as spheroids for 3 days under normoxic conditions. Later, spheroids were incubated for 24 h under hypoxia with DMSO control or 5 *μ*M Fluphenazine. Gene expression level for ATF4 target genes PPP1R15A and DDIT3 were determined using RT-qPCR. Ct values of each sample were normalized with the internal control RPL32 and normalized to the hypoxia lipid only control sample. Bars show mean with S.D. (*n*=3). (**d**) siRNA-treated cells grown as spheroids (for ATF4) or spheroids from HCT116 cells stably transfected with HIF shRNA (see Materials and Methods) were treated with either DMSO control or Fluphenazine (3 *μ*M) (or 100 *μ*M *N*-Palmitoyl-D-Sphingomyelin (SM d18:1/16)) for 3 days under hypoxia. Spheroid nuclei were stained with Hoechst (red) and dead cells were stained with SytoxGreen (green). ATF4 ameliorates Fluphenazine- or SM-induced cell death under hypoxia. *n*=3. Scale bar 100 *μ*m. ** = *P*-value between 0.001 and 0.01, *** = *P*-value between 0.0001 and 0.001

As expected, among 559 significantly upregulated transcripts (see Supplementary Figure S6) several known HIF-1-*α* target genes (Supplementary Table S4) were identified. Additionally, many central components of the ATF4- and CHOP-dependent pro-apoptotic stress-response pathway such as *ATF4*, *DDIT3* (CHOP), *PPP1R15A* (GADD34), *ATF3*, *HERPUD1* and *GADD45A*^45, 46, 47, 48^ were among the hits that show the highest upregulation after Fluphenazine–DFO co-treatment (see Figure 6a).^15^ We confirmed these results in hypoxic tumor spheroids (Figure 6b), which show a strong hypoxia-specific upregulation of ATF4 pro-apoptotic target genes *PPP1R15A* (GADD34) and *DDIT3* (CHOP) upon Fluphenazine treatment. Taken together, we conclude that Fluphenazine treatment induces the transcription of pro-apoptotic cellular stress-response genes.

Hypoxia or cellular stress pathways such as the HIF- or ATF4/CHOP-dependent pathway have been shown to activate pro-survival as well as pro-apoptotic pathways, which are balanced to determine cellular fate.^15, 47, 48, 49^ Therefore, we investigated the contribution of ATF4 or HIF signaling pathways to the induction of cell death in hypoxic spheroids. For this, we knocked down ATF4, HIF-1-*α* or HIF-2-*α* and tested whether this was sufficient to rescue Fluphenazine-induced cell death (for knockdown efficacies, see Supplementary Figure S7). Knockdown of ATF4 significantly reduced Fluphenazine-mediated overactivation of ATF4 pro-apoptotic target genes *DDIT3* (CHOP) and *PPP1R15A* (GADD34) under hypoxia (Figure 6c). Additionally, knockdown of ATF4 but not HIF-1-*α* or HIF-2-*α* partially rescued Fluphenazine- or SM-induced cell death in hypoxic spheroids (Figure 6d). This indicates that hypoxia-specific cell death induced by Fluphenazine is dependent on ATF4.

## Discussion

We here used a tumor spheroid-based assay to mimic tumor hypoxia and to screen for compounds that specifically kill cells in hypoxic tumor spheroid regions. This approach was validated by the identification of the GLUT inhibitor Cytochalasin B as well as the glycolysis inhibitor 2-deoxyglucose as hypoxia-specific inducers of spheroid cell death. Next to oxidative phosphorylation, glycolysis is one of the two main energy-producing pathways in cells. In hypoxic tumor regions, respiration is suppressed by the lack of oxygen and cells are rendered dependent on glycolysis for energy production and survival. Although inhibitors of glycolytic flux have been proposed as potential approach for cancer therapy, high toxicity in highly glucose-dependent tissues such as the brain, retina or testes have been identified as a major challenge.^9^ Therefore, we concentrated our studies on the remaining hypoxia-sensitizing screening hits that displayed a phenotype different from GLUT or glycolysis inhibitors and induced hypoxia-specific cell death by a potential novel mode of action.

Interestingly, all of these compounds showed ‘cationic amphiphilic' structural features characteristic for compounds that accumulate in lysosomes as a consequence of protonation of their basic center in the acidic lysosome environment.^30^ Indeed, Fluphenazine, one of the most potent hypoxia-selective compounds identified, leads to the accumulation of lysosomes and impairs lysosomal functions such as phospholipid metabolism by interfering with ASMase activity. However, Fluphenazine acts differently than known inducers of lysosomal dysfunction, such as Siramesine or Bafilomycin A1. These compounds induce cytotoxicity in spheroids and 2D cell culture independently of oxygen levels and cause LMP, which could be the reason for their broader cytotoxic activity and hypoxia-independent killing of cancer cells. Therefore, we speculate that Fluphenazine functionally inhibits ASMase without inducing LMP.

The main function of ASMase in lysosomes is to convert SMs to ceramide and PC. Supporting the identification of Fluphenazine as functional ASMase inhibitor, metabolomics analysis of Fluphenazine-treated cells showed a strong accumulation of SMs. Importantly, exogenous addition of SM to the cell culture medium phenocopies the effect of Fluphenazine in hypoxic spheroids. These findings support the hypothesis that SM-induced lipid stress is the main cause for the observed hypoxia-specific cell death and therefore suggests SM metabolism as potential target for the therapy of hypoxic tumors. However, cationic amphiphilic drugs such as Fluphenazine are known to alter the biophysical properties of cellular membranes and therefore could also affect other pathways, especially at the high concentrations used in this study. Accordingly, owing to the general compound toxicity we were not able to achieve concentrations of Fluphenazine or Trifluoperazine *in vivo* that would be sufficient to reach concentrations required to induce cell death in hypoxic tumor regions (data not shown).^50^

**Figure 7:**
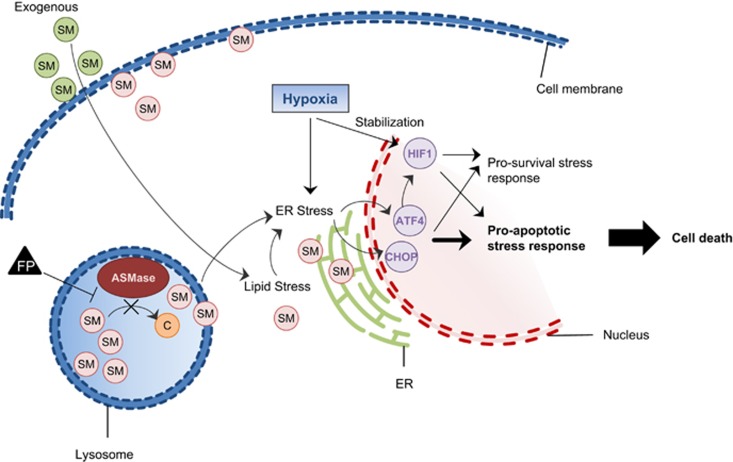
Model for Fluphenazine-induced hypoxia-specific cell death by potentiating the pro-apoptotic path of cellular stress-response pathways. Fluphenazine impairs lysosomal functions by interfering with ASMase activity. Sphingolipid accumulation induces ATF4 and CHOP. Incubation under hypoxia induces additional stress response and additionally activates HIF1 transcriptional activity. Both transcription factors, ATF4 and HIF1, express pro-survival as well as pro-apoptotic genes that must be balanced to determine cellular fate depending on the amount of stress received. Together, Fluphenazine and hypoxia treatment shift the cells' stress response toward apoptosis and cell death. However, either of these treatments alone is not sufficient to induce cell death and favors the pro-survival stress-response pathway. FP, Fluphenazine; C, ceramide; ASMase, acid sphingomyelinase; ER, endoplasmic reticulum, SM, sphingomyelin

Alterations in cellular membrane lipid compositions, for example, caused by impaired SM metabolism are known to induce lipid stress, leading to activation of ER-stress pathways, including ATF4 or XBP1 signaling.^51^ By deep sequencing, we identified the upregulation of ATF4 transcripts and ATF4 target genes after Fluphenazine treatment. Interestingly, activation of ER-stress pathways is also one of the main cellular responses to hypoxia.^9^ On the other hand, hypoxia also leads to the stabilization of HIF transcription factors. These signaling pathways are partially overlapping^12, 44, 52, 53^ and are balanced to either mediate a pro-survival or a pro-apoptotic response, depending on the severity of stress received.^14, 47, 48, 54, 55^ Fluphenazine treatment or SM supplementation lead to an overinduction of the hypoxia response, including both HIF and ATF4 transcriptional overactivity, specifically in HIF-stabilized cells or hypoxic spheroids. Therefore, a possible explanation for the observed hypoxia-specific induction of cell death in spheroids could be that simultaneous induction of hypoxia- and lipid-stress pathways lead to alterations in hypoxia-stress signaling, tipping the balance from a pro-survival to a pro-apoptotic stress response (see Figure 7). Interestingly, we find that cell death induction in hypoxic spheroids by Fluphenazine is not dependent on HIF but on ATF4 transcriptional activity.

Based on these findings, we propose a model in which Fluphenazine induces cellular SM accumulation by functionally inhibiting ASMase. Accumulation of SM in turn induces lipid stress signaling by ATF4.^51^ Combining both, lipid stress and hypoxic stress, shifts the balance of cellular stress signaling to an ATF4-dependent pro-apoptotic response and drives cells into apoptosis specifically in highly stressed hypoxic or anoxic tumor cells (see Figure 7).

So far, most approaches to target hypoxic tumor cells have focused to a great extent on the inhibition of hypoxia-stress pathways.^16^ This has been owed to the fact that HIF-1 is overexpressed in a large number of human tumors and correlates with poor prognosis and treatment failure.^56^ Nevertheless, to date there is no clear clinical evidence of antitumor activity due to HIF-1 inhibition^9^ and no specific HIF-1 inhibitor has been clinically approved.^8^ In contrast, the here presented data suggest a novel, yet unexplored, mechanism in which induction of lipid stress could potentially lead to overactivation of hypoxia-stress-response pathways and thereby promote their pro-apoptotic tumor-suppressor functions to specifically kill cells in hypoxic tumor areas.

## Materials and Methods

### Cell culture

All cell lines were obtained from American Type Culture Collection (Manassas, VA, USA). HCT116 and T47D were cultured in RPMI 1640 (Gibco) Supplemented with 10% FCS (PAA Laboratories by GE Healthcare, Little Chalfont, UK) and 1% Penicillin/Streptomycin (Sigma-Aldrich, St. Louis, MO, USA) (and 0.01 *μ*g/ml Insulin for T47D cells (Gibco by Thermo Fisher, Waltham, MA, USA)). HCT116 cells carrying the HRE-Luciferase reporter construct (598-pGL3-Hif-RE-Luc vector)^41^ were cultured in DMEM (Gibco) supplemented with 10% FCS, 1% PS and 100 *μ*g/ml Hygromycin B (Invitrogen, Carlsbad, CA, USA). Cells were maintained at 37 °C in a 5% CO_2_ and 95% air incubator. Cells were subjected to hypoxia by exposure to <1% O_2_, 5% CO_2_ and balance N_2_ at 37 °C in an incubator chamber (C16, Labotect, Rosdorf, Germany).

### Immunohistochemistry: HCT116 tumor section

1.5 × 10^6^ human colon cancer HCT116 cells were injected subcutaneously in cell medium into the left flank of female NMRI nude mice (Charles River, Wilmington, MA, USA) to establish a subcutaneous tumor. At a tumor size of an average of 100 mm^2^, the animals were intravenously injected with Pimonidazole (Hypoxyprobe, Burlington, MA, USA) according to the manufacturer's instructions and killed 1 h later; the tumors were resected and fixed in 10% formalin. The tumors were fixed in formalin for 48 h and further processed in an Autotechnicon (Leica ASP 200S, Leica Biosystems, Wetzlar, Germany), embedded in paraffin (Microm EC350-1, Thermo Fisher, Waltham, MA, USA), sliced and generated sections were transferred to object slides. Animal experiments were conducted in accordance with the German animal welfare law, approved by local authorities and in accordance with the ethical guidelines of Bayer AG.

Prior to immunostaining, slides were deparrafined using a decreasing alcohol dilution series. Later, heat-mediated antigen retrieval was performed and unspecific binding sides were reduced using Protein block (Dako by Agilent Technologies, Santa Clara, CA, USA). Vasculature was stained using a 1:100 dilution of anti-CD31 (ab28364)) and 1:100 fluorescent Alexa 546 anti-rabbit secondary antibody (Jackson ImmunoResearch, West Grove, PA, USA). Hypoxia was detected via bound pimonidazole adducts using a 1:100 mouse–anti-pimonidazole–FITC (Hypoxyprobe, HP6-100Kit). Additionally, tissue sections were stained with Hoechst 33342 (Life Technologies by Thermo Fisher, Waltham, MA, USA) to label cell nuclei. Slides were imaged for fluorescence on the ImageXpress Micro widefield imaging system (Molecular Devices, Sunnyvale, CA, USA) with 10 × air objective and attached CCD camera.

### Spheroid generation

Spheroid generation was carried out using a modified version of the liquid overlay cultivation technique described previously.^57^ Briefly, 10 *μ*l of a heated 1.5% (w/v) agarose (in RPMI 1640, no FCS) solution was dispended by a liquid dispenser (Multidrop Combi, Thermo Fisher, Waltham, MA, USA) into sterile 384-well clear bottom imaging plates (Greiner, Kremsmuenster, Austria). For spheroid growth, a single-cell suspension was seeded into agarose-coated 384-well clear bottom plates in 40 *μ*l culture medium using a liquid dispenser. Plates were incubated under standard cell culture conditions at 37 °C and 5% CO_2_ in humidified incubators for 4 days. To obtain spheroids with an approximate diameter of 400 *μ*m on day 4, 300 cells were seeded per well for HCT116 and 2000 cells per well for T47D. This setup led to the formation of one spheroid per well with high intra-well and intra-plate reproducibility concerning spheroid area and roundness (Supplementary Figure S1).

### Immunohistochemistry: Spheroids

Three hours prior to harvest, spheroids were incubated with 100 *μ*M pimonidazole (Hypoxyprobe) under previous culture conditions. After 2 h pimonidazole incubation, spheroids were fixed for 1 h in 4% PFA. Later, spheroids were transferred to 50 ml tubes, washed in DPBS (Dulbecco's Phosphate Buffered Saline) and equilibrated in 30% sucrose/ 5% glucose (w/v) DPBS solution for 1 h. Then spheroids were transferred to cryomolds, excess DPBS solution was removed and spheroids were covered in Tissue-Tek OCT compound (Sakura, Staufen, Germany). After equilibration, cryomolds were frozen by incubation in a mixture of dried ice and 2-Methylbutane (Sigma-Aldrich). Prepared samples were cut into 5 *μ*m sections by cryostat and mounted on SuperFrost Plus slides (Menzel-Glaser, Braunschweig, Germany). Visualization of hypoxic areas was carried out using a mouse FITC-MAb against pimonidazole (Hypoxyprobe). Furthermore, sections were counterstained with Hoechst 33342 (Life Technologies) to stain nuclei. Slides were imaged on the ImageXpress Micro widefield imaging system (Molecular Devices) with 10 × air objective and attached CCD camera.

### Compound treatment

After 4 days of spheroid growth, 20 *μ*l culture medium containing 80 nl compounds (ENZO Screen-Well ICCB Known Bioactives library, Enzo Life Sciences, Farmingdale, NY, USA (468 compounds), final compound dilution of 0.1–20 *μ*M, depending on original stock concentration) were added and incubated for additional 3 days either at normal culture conditions (21% O_2_, 37 °C, 5% CO_2_) or in a hypoxic chamber (<1% O_2_, 37 °C, 5% CO_2_). A 0.2% DMSO solution was used as solvent control and Staurosporine (Sigma-Aldrich) as general toxic control (10 *μ*M).

Screening hits and further tool compounds, including Staurosporine, Antimycin A, 2-DG, Fluphenazine, Chlorpromazine, Thioridazine, Clozapine, Bafilomycin A, Siramesine, *N*-Palmitoyl-D-sphingomyelin, SM (from bovine brain), Ceramide (from bovine spinal cord) and 2-Dioleoyl-sn-glycero-3-phosphocholine (18:1 (Δ9-Cis) PC), were purchased from Sigma-Aldrich. 1-Stearoyl-2-arachidonoyl-sn-glycero-3-phosphocolin (18:0–20:4 PC) and 1-stearoyl-2-docosahexaenoyl-sn-glycerol-3-phosphocolin (18:0–22:6 PC) were purchased from Avanti Lipids (Alabaster, AL, USA). All compounds, except for SM and PCs, were dissolved in DMSO (10 mM) and stored at −20 °C. SM and PCs were dissolved in ethanol. Hypoxia mimicking agent DFO (Sigma-Aldrich) was used at a final concentration of 1 mM (2D growth assays for 16–24 h).

### Image acquisition and analysis of spheroids

Prior to imaging, spheroids were stained for a minimum of 2 h by adding Hoechst 33342 (Life Technologies) as counterstain for all nuclei and SytoxGreen as stain for dead cells (Life Technologies) at a final dilution of 1:10 000 each. This set up led to a high intra-well and intra-plate reproducibility concerning spheroid size and dead cell staining (Supplementary Figure S1).

One image per spheroid and wavelength, focused on the spheroid center, was captured by an Opera confocal spinning disc microscope system with a 4 × air objective. Quantification of spheroid cell death was carried out with the MetaXpress software (Molecular Devices) using custom written image analysis routines as described previously.^57^

Normalization, quality control and fitting curves for EC50 determination of tested compounds were carried out with Genedata Screener for high-content screening and Genedata Condoseo modules (Genedata AG, Basel, Switzerland). In detail, wells with no recognizable spheroid were masked and the average intensity of the dead cell signal (SytoxGreen) was normalized to the DMSO control (0%) and the 10 *μ*M Staurosporine (100%) control.

The pilot screen performance was characterized by a robust RZ factor of 0.65.

### Real-time quantitative PCR

Total RNA was isolated from HCT116 cells or spheroids using RNeasy Plus Mini Kit (Qiagen, Venlo, Netherlands) and reverse-transcribed with the RevertAid H Minus First Strand cDNA Synthesis Kit (Thermo Fisher) according to the manufacturer's instructions. To measure the expression levels of target genes, sample concentrations were adjusted to 10 ng/*μ*l cDNA and mixed with specific TaqMan Gene Expression Primer (Thermo Fisher) and TaqMan Fast Advanced Master Mix (Thermo Fisher). Real-time quantification was performed in quadruplicates on a MicroAmp optical 384-well reaction plate (Thermo Fisher) using a 7900 PCR machine (Applied Biosystems by Thermo Fisher, Waltham, MA, USA). Relative mRNA levels were calculated to the geometric mean of reference gene RPL32 (encoding ribosomal protein L32).

TaqMan Primers used were: RPL32 (ribosomal protein L32. Hs00851655_g1), SLC2a3 (solute carrier family 2 (facilitated glucose transporter), member 3, encoding Glut3 protein, Hs00359840_m1), VEGFA (vascular endothelial growth factor A, Hs00900055_m1), BNIP3 (BCL2/ adenovirus E1B 19kDa interacting protein 3, Hs00969291_m1), PPP1R15A (Protein phosphatase 1 regulatory subunit 15A, Hs00169585_m1), DDIT3 (DNA-damage-inducible transcript 3, Hs99999172_m1), ATF4 (Activating transcription factor 4, Hs00909569_g1), HIF-1-*α* (Hypoxia inducible factor 1 alpha, Hs00153153_m1), and EPAS1 (Endothelial PAS domain-containing protein 1, Hs01026149_m1).

### Lipidomics analysis

After compound treatment for 24 h at 37 °C and 21% O_2_ (*n*=3), HCT116 cells were washed twice with cold sodium chloride (0.9%) and incubated for 15 min with 1 ml 80% methanol at −80 °C. Subsequently, cells were harvested using a cell scraper and transferred together with the methanol into a new tube. Wells were washed once with 500 *μ*l 80% methanol and added into the same tube. The resulting extracts were freeze-dried and resolved in 100 *μ*l 100% methanol followed by a centrifugation step. Later, 10 *μ*l of these extracts were used for target metabolite profiling by using the LC-MS based AbsoluteIDQ p180 Kit (Biocrates Life Sciences AG, Innsbruck, Austria). All samples were processed according to the manufacturer's instruction, and measurements were performed with a UHPLC-MS/MS System (Shimadzu UHPLC, Shimadzu, Kyoto, Japan and Sciex 5500 mass spectrometer, Sciex, Framingham, MA, USA). Multivariate data analysis was carried out by using the Umetrics SIMCA-P software (MKS Data Analytics Solutions, Malmö, Sweden).

### Deep sequencing

HCT116 cells were seeded in 12-well plates and treated for 24 h with either Fluphenazine (5 *μ*M) alone or with Fluphenazine+DFO (1 mM) at 37 °C and 21% O_2_ (*n*=4). Total RNA of each sample was extracted using the RNeasy Plus Mini Kit (Qiagen) according to the manufacturer's instructions. Subsequently, the TruSeq RNA Sample Preparation Kit v2 (Illumina, San Diego, CA, USA) was used to convert the mRNA of each sample into a library of template molecules for DNA sequencing. Briefly, poly-A containing mRNA molecules were purified using oligo-dT attached magnetic beads. This was followed by mRNA fragmentation using divalent cations and subsequent first-strand (reverse transcriptase and random primers) and second-strand cDNA synthesis (DNA Polymerase I and RNase H). The synthesized cDNA fragments then went through an end repair process, the addition of a single ‘A' base and ligation of the adapters.

Library quality was evaluated using the Agilent DNA 1000 Chip Kit (Agilent Technologies, Santa Clara, CA, USA) and DNA quantity was determined using the KAPA Library Quantification Kit (Kapa Biosystems, Wilmington, MA, USA) according to the manufacturer's instructions. All libraries were pooled and adjusted to a final concentration of 10 nM before they were sequenced according to standard protocols for the Illumina HiSeq 2500. Briefly, using the HiSeq SBS Reagent Kit v4 the sample cDNA library was denatured, mixed with HT1 and adjusted to 18 pM. The sample library was then mixed with a PhiX Library and applied to a HiSeq v4 flow cell (Illumina), which was subsequently clustered on a cBot (Illumina) using the HiSeq v4 PE Cluster Kit (Illumina) before all samples were sequenced in the HiSeq 2500 system. On average 39±6 million clusters were sequenced per sample. Reads were mapped to the human genome (version hg19) using STAR aligner (version 2.4.2) and read counts were assigned to 20776 annotated genes (gencode v19).

Statistical analysis was performed using the R statistical programming environment version 3.1.2. The standard workflow implemented in the DESeq2^58^ package version 1.6.3 was employed for identifying differentially expressed genes between samples treated with DFO and FP compared with samples treated only with DFO. Genes not expressed in any sample were removed before analysis. Genes were defined as differentially expressed if they showed a fold change >2 in either of the conditions and had a Benjamini–Hochberg corrected *P*-value <0.05. Fisher's Exact test was used to identify Hallmark gene signatures from the Molecular Signatures Database^59^ version 5.1 overlapping with the list of genes significantly upregulated with treatment of DFO+FP compared with treatment with DFO alone. Hallmarks with a Benjamini–Hochberg corrected *P*-value <0.05 were regarded as significantly overlapping.

### Western blotting

Protein levels of HIF-1-*α* in HCT116 under various treatment conditions were obtained via western blotting. Briefly, HCT116 cells were either seeded in 2D in 6-well plates or on 384-well agarose-coated plates for spheroid formation (4 days). After overnight incubation (2D) or 4 days of spheroid growth, cells were treated with the compounds (DMSO control, Fluphenazine 5 *μ*M) under different conditions (Normoxia, Hypoxia or Normoxia+1 mM DFO). After treatment, cells or spheroids were collected, lysed for 10 min with cold lysis buffer (0.5 M Tris-HC, pH 7.4, 1.5 M NaCl, 2.5% deoxycholic acid, 10% NP-40, 10 mM EDTA; Merck Millipore, Billerica, MA, USA) containing protease and phosphatase inhibitor (Thermo Fisher). The lysate was centrifuged for 10 min at 4 °C (14 000 r.p.m.), and the supernatant was collected for determining the protein content of each probe using the Pierce BCA Protein Assay Kit with a BSA standard (Thermo Fisher) according to the manufacturer's instructions. Proteins were separated using 4–12% Bis-Tris gels (Invitrogen) according to the manufacturer's instructions. Later, the separated proteins were transferred to nitrocellulose membranes (Life Technologies) using an iBlot gel transfer device (Life Technologies). The membranes were blocked for 1 h in 5% milk and later washed with 1 × TBST-Buffer (Carl Roth, Karlsruhe, Germany).

Following the blocking procedure, blots were incubated overnight at 4 °C with the primary antibodies, anti-HIF1-a (Abcam ab51608, Cambridge, UK) 1:200 and anti-beta-actin (Sigma-Aldrich A5316) 1:3000. For quantification, blots were washed with TBST and the membranes were incubated with an IRDye 680RD-anti-mouse IgG (Licor, Lincoln, NE, USA) or an IRDye 800CW-anti-rabbit IgG (Licor). Blots were imaged using an Odyssey CLx Imaging System (Licor). Quantification of bands was performed using Image Studio Lite Ver. 4 (Licor).

### Immunofluorescence

After formaldehyde fixation with 4% PFA, cells were permeabilized with 0.1% Triton-X100 (Sigma-Aldrich) and unspecific binding sides were blocked using 1% BSA. Mouse anti-Lamp2 (Santa Cruz Biotechnology sc-18822, Dallas, TX, USA, 1:200) and rabbit anti- Galectin 1 (Abcam ab25138, 1:250) were used as primary antibodies and appropriate secondary antibodies conjugated with Alexa-Fluor 488 (Jackson ImmunoResearch) were used. Cell nuclei were stained with Hoechst 33342 (Life Technologies). Images were acquired by an Opera confocal spinning disc microscope system with a 40 × water objective. Quantification of Galectin or Lamp2 staining (granules per cell and/or granules intensity) was carried out with the MetaXpress software (Molecular Devices).

### LipidTOX Phospholipidosis assay

To visualize phospholipid accumulation in lysosomes, the LipidTOX Red Phospholipidosis Detection Reagent (Thermo Fisher, 1:2000) was added to the cells with compound treatment according to the manufacturer's instructions. After 24 h incubation at standard cell culture conditions, Hoechst 33342 (Life Technologies) was added to the cells at a final dilution of 1:5000. Images were acquired using the ImageXpress Micro widefield imaging system (Molecular Devices) with a × 40 magnification. Quantification of phospholipid accumulation (granules per cell and/or granules intensity) was carried out with the MetaXpress software (Molecular Devices).

### Lysotracker assay

After 24 h compound treatment, LysoTracker Red DND-99 (Thermo Fisher) and Hoechst 33342 (Life Technologies) were added to the cells at a final dilution of 1:2000 and 1:5000. Cells were incubated with the staining reagent for 30 min at 37 °C before they were imaged using the ImageXpress Micro widefield imaging system (Molecular Devices) with a × 40 magnification. Quantification of lysosomes (granules per cell and/or granules intensity) was carried out with the MetaXpress software (Molecular Devices).

### ASMase assay

After 24 h compound treatment, HCT116 cells were lysed by performing multiple freeze–thaw cycles. The lysate was centrifuged for 10 min at 4 °C (14 000 r.p.m.) and the supernatant was collected for determining the protein content of each probe using the Pierce BCA protein assay kit with a BSA standard (Thermo Fisher) according to the manufacturer's instructions. Subsequently, the activity of ASMase was measured using the Echelon Acid Sphingomyelinase Assay Kit according to the manufacturer's instructions. Briefly, 20 *μ*g per 20 *μ*l sample or diluted standards were added to a 96-well plate and mixed with 30 *μ*l substrate buffer. Afterwards, 50 *μ*l diluted fluorogenic ASMase-specific substrate were added, and the plate was incubated at 37 °C for 3 h with shaking before it was further diluted and incubated for 10 min with 50 *μ*l stop buffer. Subsequently, fluorescence levels were determined using a Tecan Plate reader (Tecan Trading AG, Männedorf, Switzerland).

### BODIPY FL C12-Sphingomyelin

To visualize cellular SM uptake and localization, the fluorescent reagent BODIPY FL C12-Sphingomyelin (Thermo Fisher, 1 mM) was added to the cells with compound treatment. After 24 h incubation at standard cell culture conditions, Hoechst 33342 (Life Technologies) was added to the cells at a final dilution of 1:5000. Additionally, lysosomes were stained using LysoTracker Red DND-99 (Thermo Fisher, 1:2000). Images were acquired by an Opera confocal spinning disc microscope system with a 40 × water objective.

### HRE-Luciferase reporter assay

To study HIF signaling, a HCT116 reporter cell line was used that is transfected with a 598-pGL3-HIF-RE-Luc reporter plasmid that contains a luciferase gene expressed under the control of a VEGF promoter-derived HRE.^41^ Cells were selected using 100 *μ*g/ml Hygromycin B (Invitrogen). Luciferase activity was measured using the luminescence kit Steady-Glo (Promega, Fitchburg, WI, USA) and a luminescence plate reader (PHERAstar (BMG Labtech, Ortenberg, Germany)) according to the manufacturer's instructions. Briefly, HCT 116-HRE-Luc cells were plated at 3000 cells/5 *μ*l in white 384-well small volume plates (Greiner) using a liquid dispenser (Multidrop Combi, Thermo Scientific), 5 *μ*l compounds were added and cells were either incubated in a normal incubator or in a hypoxia chamber at >1% O^2^ for 16 to 24 h. In all, 5 *μ*l SteadyGlo (Promega) were then added to cells and incubated for 30 min in the dark. Subsequently, luminescence intensity was measured in a plate reader.

### siRNA and shRNA transfection

To generate ATF4 knockdown cells, HCT116 cells were incubated with ATF4 siRNA (Thermo Fisher s1703, 10 nM) and Lipofectamine RNAiMAX (Thermo Fisher, 1:1000) lipid or control (lipid only) in agarose-coated 384-well plates and grown as spheroids for 3 days at 37 °C and 21% O_2_ (see ‘Spheroid generation'). To generate HIF-1-*α* and EPAS1 knockdown cells, HCT116 cells were stably transfected with shRNA (Sigma-Aldrich, TRCN0000318674 and TRCN0000003803). Cells were selected using 0.6 *μ*g/ml Puromycin (Sigma-Aldrich) and subsequently grown to spheroids in agarose-coated 384-well plates for 4 days at 37 °C and 21% O_2_.

### Statistical analysis

For statistical analysis, the Prism Scientific Presentation Software (GraphPad Software, La Jolla, CA, USA) was used. Values were compared by applying a two-tailed *t*-test with Welch's correction. The statistical significance between two levels is represented by asterisks (*P-value between 0.01 and 0.05; **P-value between 0.001 and 0.01; ***P-value between 0.0001 and 0.001 and ****P-value <0.0001). Error bars in graphs represent S.D.

## Figures and Tables

**Table 1:**
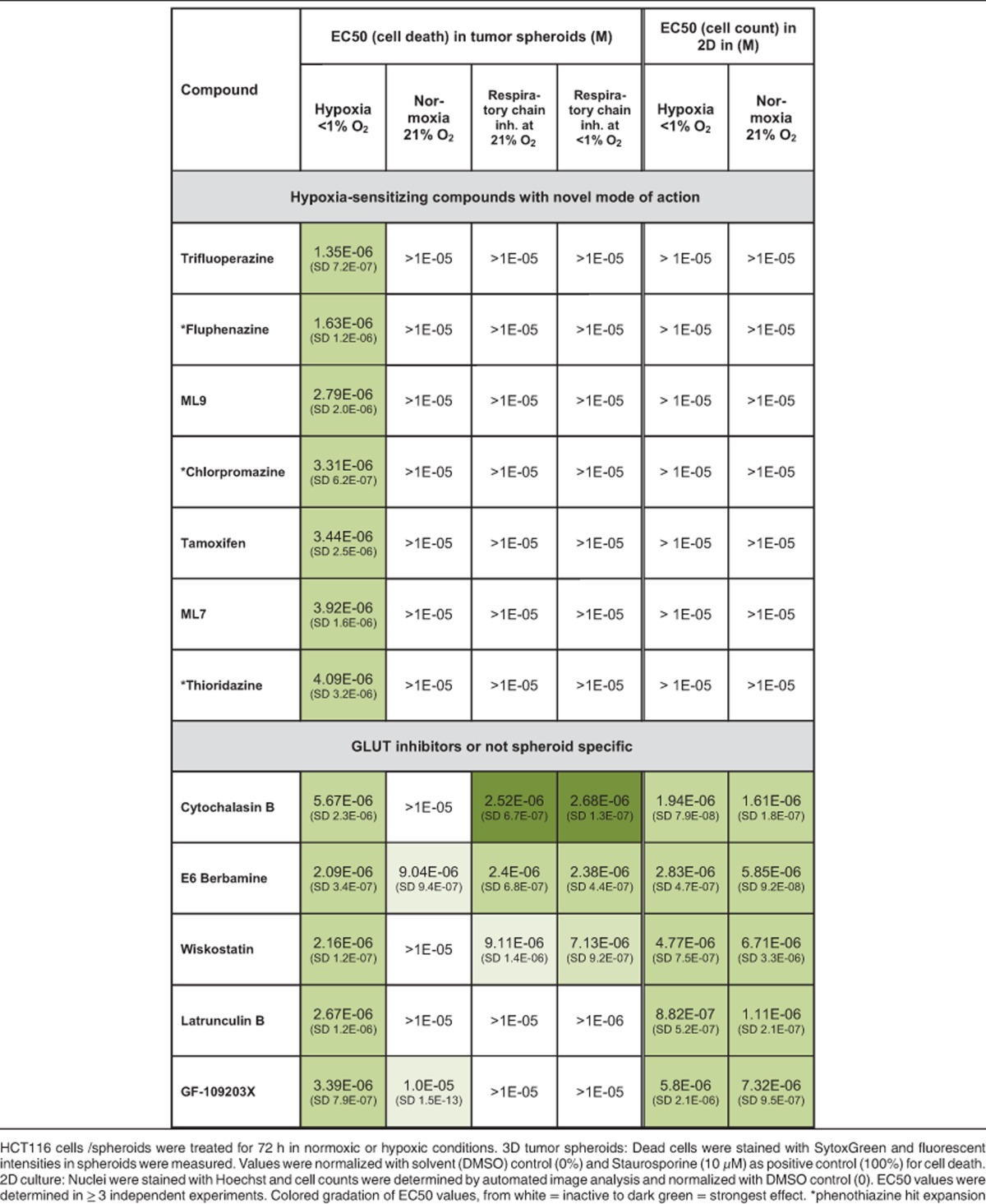
Retest and EC50 generation of hypoxia specific hits in hypoxic and normoxic 3D tumor spheroids (without or with respiratory chain inhibitor Antimycin (200 nM)) or in 2D culture (HCT116)

**Table 2:**
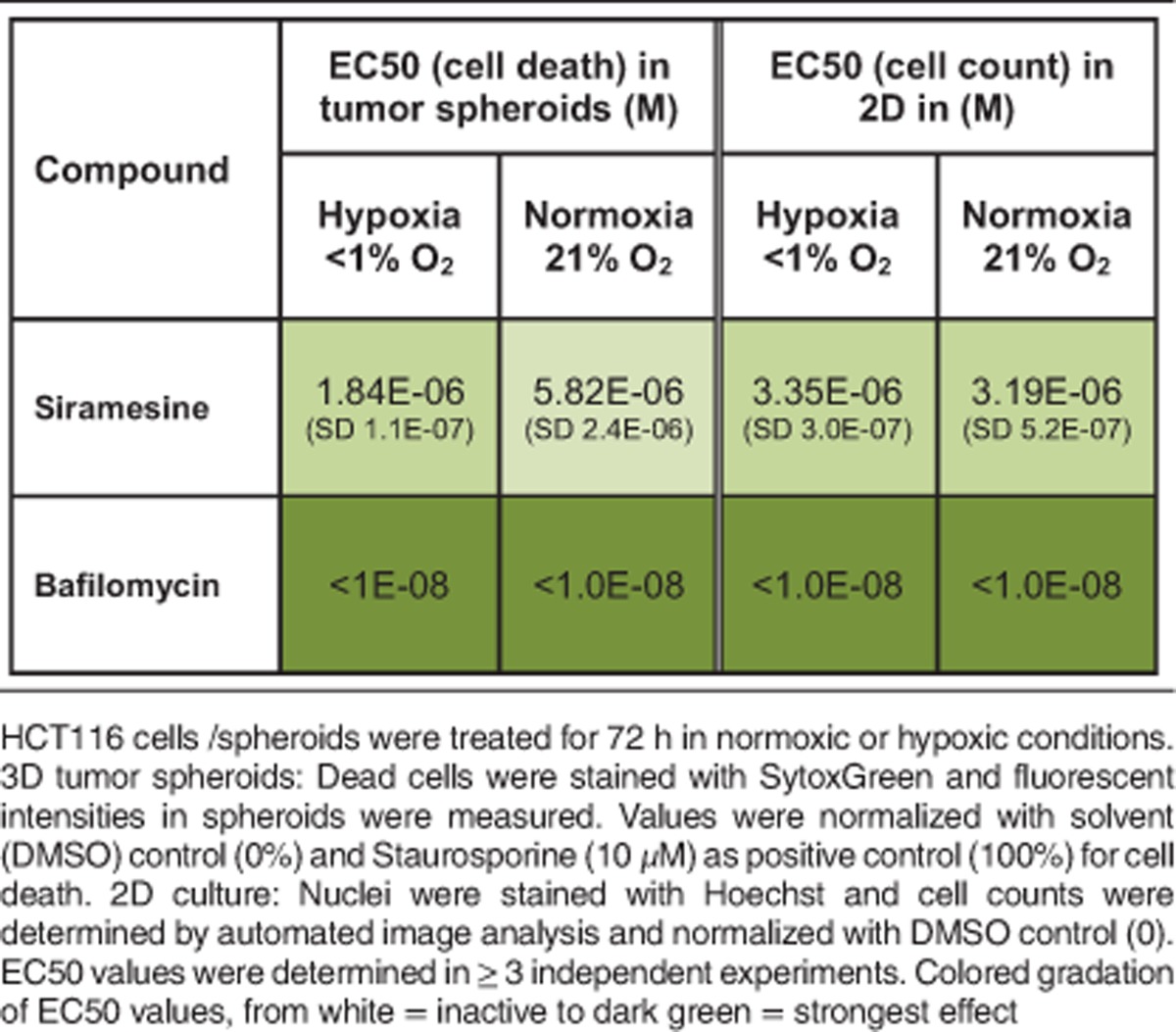
Siramesine and Bafilomycin A1 induce tumor spheroid cell death independently of oxygen levels
