# Supplementary material for: Functional inhibition of acid sphingomyelinase by Fluphenazine triggers hypoxia-specific tumor cell death
Source: Cell Death Dis. 2017 Mar 30;8(3):e2709–. doi: 10.1038/cddis.2017.130 (PMC5386533; doi:10.1038/cddis.2017.130)
Supplement: Supplementary Tables [file cddis2017130x1.docx]

**Supplementary Table 1: Complete compound list of phenotypic screen in hypoxic vs. normoxic HCT116 tumor spheroids.** Dead cells were stained with SytoxGreen and fluorescent intensities in spheroids were measured. Values were normalized with solvent (DMSO) control (0%) and Staurosporine (10 µM) as positive control (100%).

| **Compound** | **Concen-tration [M]** | **Repli-cates** | **Hypoxia** | | **Normoxia** | | **Ratio: Hypoxia/ Normoxia** |
| --- | --- | --- | --- | --- | --- | --- | --- |
|  |  |  | **Normalized Average Intensity** | **Std Dev** | **Normalized Average Intensity** | **Std Dev** |  |
| **DMSO** | - | 181 | **0** | 0.23 | **0** | 0.16 | 0.0 |
| **Staurosporine control** | 1.0E-05 | 91 | **100** | 0.87 | **100** | 1.47 | 1.0 |
| (-)11(12)-Epoxyeicosatrienoic acid | 1.3E-07 | 1 | **-3.11** | - | **-0.39** | 0.36 | 7.9 |
| (-)14,15-Epoxyeicosa-5Z,8Z,11Z-trienoic acid | 1.3E-07 | 2 | **-3.2** | 2.81 | **0.16** | 0.6 | -19.8 |
| (-)-Epibatidine | 3.2E-05 | 4 | **2.37** | 1.61 | **6.1** | 2.48 | 0.4 |
| (-)-Huperzine A | 2.8E-05 | 4 | **1.36** | 3.85 | **4.27** | 3.07 | 0.3 |
| (R)-(+)-BAY K-8644 | 1.9E-05 | 4 | **4.28** | 0.84 | **1.81** | 0.74 | 2.4 |
| (R)-(+)-Methandamide | 1.3E-06 | 2 | **-4.03** | 2.19 | **-1.22** | 0.46 | 3.3 |
| (S)-(-)-propranolol-HCl | 2.3E-05 | 4 | **28.3** | 8.18 | **0.4** | 0.55 | 70.1 |
| 1,25-Dihydroxyvitamin D3 | - | 1 | **-5.72** | - | **-3.25** | 0.32 | 1.8 |
| 1,2-Didecanoyl-glycerol (10:0) | 1.3E-06 | 1 | **-0.33** | - | **0.71** | 0.11 | -0.5 |
| 1,2-Dioctanoyl-SN-glycerol | 1.3E-06 | 1 | **-2.65** | - | **0.72** | 0.85 | -3.7 |
| 1,2-Dioleoyl-glycerol (18:1) | 1.3E-06 | 0 | **-** | - | **0.12** | 1.18 | - |
| 10-hydroxycamptothecin | 1.8E-05 | 4 | **118.64** | 7.23 | **116.24** | 12.7 | 1.0 |
| 12(R)-HETE | 1.3E-07 | 1 | **-11.81** | - | **-0.3** | 0.36 | 38.7 |
| 12(S)-HETE | 1.3E-07 | 2 | **-4.7** | 0.48 | **-0.71** | 0.15 | 6.6 |
| 12(S)-HPETE | - | 0 | **-** | - | **-0.54** | 0.83 | - |
| 12-Methoxydodecanoic acid | - | 0 | **-** | - | **-1.23** | 0.1 | - |
| 13(S)-HODE | 1.3E-07 | 2 | **-1.87** | 3.52 | **-1.21** | 0.86 | 1.5 |
| 13(S)-HPODE | 1.3E-07 | 2 | **-1.99** | 2.56 | **-0.4** | 0.81 | 4.9 |
| 13,14-Dihydro-PGE1 | 1.3E-06 | 2 | **-4.5** | 1.08 | **-0.7** | 0.71 | 6.4 |
| 13-cis retinoic acid | 1.3E-06 | 2 | **1.11** | 1 | **0.16** | 0.58 | 7.0 |
| 13-Keto-octadeca-9Z,11E-dienoic acid | 1.3E-07 | 3 | **-4.36** | 5.89 | **0.52** | 0.63 | -8.4 |
| 1400W-2HCl | 2.7E-05 | 4 | **2.55** | 1.3 | **-0.05** | 0.79 | -53.7 |
| 15(S)-HETE | 1.3E-07 | 2 | **-3.56** | 1.15 | **-0.11** | 0.11 | 32.4 |
| 15(S)-HPETE | 1.3E-07 | 2 | **-1.7** | 2.42 | **0.57** | 0.7 | -3.0 |
| 15-deoxy-Prostaglandin J2 | - | 0 | **-** | - | **-0.65** | 0.19 | - |
| 15-Ketoicosatetraenoic acid | - | 0 | **-** | - | **-0.96** | 0.11 | - |
| 16,16-Dimethyl-prostaglandin E2 | - | 0 | **-** | - | **-1.58** | 0.19 | - |
| 17-Allylamino-geldanamycin | 1.1E-05 | 4 | **49.67** | 10.5 | **37.1** | 5.73 | 1.3 |
| 17-Octadecynoic acid | 1.3E-06 | 1 | **13.82** | - | **0.31** | 0.69 | 44.4 |
| 17-Phenyl-trinor-prostaglandin E2 | - | 0 | **-** | - | **1.31** | 0.74 | - |
| 1-Acyl-PAF | - | 0 | **-** | - | **0.08** | 0.65 | - |
| 1-Deoxymannojirimycin hydrochloride | 3.3E-05 | 4 | **-1.29** | 0.26 | **-1.2** | 0.3 | 1.1 |
| 1-Deoxynojirimycin | 4.1E-05 | 4 | **-0.15** | 1.66 | **-0.53** | 0.18 | 0.3 |
| 1-Hexadecyl-2-arachidonoyl-glycerol | 1.3E-06 | 1 | **-10.98** | - | **-1.41** | 0.39 | 7.8 |
| 1-Hexadecyl-2-methylglycero-3 PC | - | 0 | **-** | - | **-0.1** | 0.6 | - |
| 1-Hexadecyl-2-O-acetyl-glycerol | 1.3E-06 | 1 | **-11.73** | - | **0.21** | 0.15 | -57.0 |
| 1-Octadecyl-2-methylglycero-3 PC | 1.3E-06 | 4 | **-3.59** | 0.84 | **-0.08** | 0.49 | 42.4 |
| 1-Oleoyl 2-acetyl-glycerol | 1.3E-06 | 3 | **-3.3** | 0.59 | **-0.92** | 0.58 | 3.6 |
| 1-Stearoyl-2-lineoyl-glycerol | 1.3E-06 | 2 | **-4.38** | 0.68 | **-1.64** | 0.68 | 2.7 |
| 2,5-Ditertbutylhydroquinone | 3.0E-05 | 4 | **1.92** | 2.72 | **3.2** | 4.3 | 0.6 |
| 24(S)-hydroxycholesterol | 1.7E-05 | 4 | **18.98** | 0.27 | **20.97** | 17.2 | 0.9 |
| 24,25-Dihydroxyvitamin D3 | 1.3E-06 | 1 | **4.49** | - | **0.93** | 0.16 | 4.9 |
| 25-Dihydroxyvitamin D3 | - | 0 | **-** | - | **-0.75** | 0.19 | - |
| 2-APB | 3.0E-05 | 4 | **1.68** | 2.88 | **0.96** | 0.84 | 1.8 |
| 2-Arachidonoylglycerol | 1.3E-06 | 1 | **-0.18** | - | **0.04** | 0.75 | -4.2 |
| 2-Fluoropalmitic acid | 1.3E-06 | 1 | **-2.4** | - | **-0.62** | 0.36 | 3.9 |
| 2-Hydroxymyristic acid | 1.3E-06 | 2 | **-1.92** | 0.49 | **0.18** | 0.36 | -10.9 |
| 2-methoxyantimycin A3 | 1.3E-05 | 4 | **2.83** | 1.3 | **-1.37** | 0.25 | -2.1 |
| 3,4-dichloroisocoumarin | 3.1E-05 | 4 | **-0.09** | 0.62 | **0.74** | 0.8 | -0.1 |
| 3-aminobenzamide (3-ABA) | 4.9E-05 | 4 | **-1.4** | 1.6 | **-1.51** | 0.4 | 0.9 |
| 4-Amino-1,8-naphthalimide | 3.1E-05 | 4 | **1.11** | 0.38 | **0.26** | 0.7 | 4.3 |
| 4-Aminopyridine | 7.1E-05 | 4 | **4.99** | 2.82 | **0.41** | 0.61 | 12.1 |
| 4-hydroxyphenylretinamide | 1.3E-06 | 3 | **1.98** | 0.24 | **0.83** | 0.8 | 2.4 |
| 4-Oxatetradecanoic acid | 1.3E-06 | 4 | **-3.86** | 0.37 | **-0.89** | 0.2 | 4.3 |
| 5(S)-HETE | 1.3E-07 | 4 | **-2.08** | 0.69 | **-1.44** | 0.49 | 1.4 |
| 5(S)-HPETE | 1.3E-07 | 4 | **-3.33** | 0.21 | **-0.45** | 0.49 | 7.5 |
| 5,6-Epoxyeicosatrienoic acid | 1.3E-07 | 4 | **-1.04** | 1.26 | **-0.62** | 0.6 | 1.7 |
| 5,8,11,14-Eicosatetraynoic acid | 1.3E-06 | 4 | **-0.46** | 0.76 | **1.92** | 1.1 | -0.2 |
| 5,8,11-Eicosatriynoic acid | 1.3E-06 | 4 | **-0.78** | 0.23 | **-0.4** | 0.66 | 2.0 |
| 5-Hydroxydecanoate | 3.5E-05 | 4 | **-1.17** | 1.19 | **0.1** | 0.92 | -11.4 |
| 5-Iodotubercidin | 1.7E-05 | 4 | **7.85** | 1.53 | **15.67** | 0.72 | 0.5 |
| 5-Ketoeicosatetraenoic acid | 1.3E-07 | 4 | **-0.84** | 0.99 | **-0.81** | 0.8 | 1.0 |
| 5'-N-Ethylcarboxamidoadenosine (NECA) | 2.2E-05 | 4 | **0.97** | 0.23 | **2.95** | 0.73 | 0.3 |
| 6(5H)-Phenanthridinone | 3.4E-05 | 4 | **-1.04** | 1.36 | **5.87** | 2.48 | -0.2 |
| 6,7-ADTN | 3.7E-05 | 4 | **1.74** | 2.62 | **-1.62** | 0.03 | -1.1 |
| 6-Formylindolo [3,2-B] carbazole | 1.3E-06 | 4 | **-1.29** | 0.82 | **-0.74** | 0.36 | 1.7 |
| 6-Gingerol | 2.3E-05 | 4 | **-0.22** | 0.69 | **2.17** | 0.64 | -0.1 |
| 6-Keto-prostaglandin F1a | 1.3E-06 | 4 | **-3.16** | 0.67 | **-0.93** | 0.12 | 3.4 |
| 7,7-Dimethyleicosadienoic acid | 1.3E-06 | 4 | **0.02** | 0.7 | **-0.37** | 0.86 | -0.1 |
| 8,9-Epoxyeicosatrienoic acid | 1.3E-07 | 4 | **-1.85** | 0.2 | **-0.64** | 0.9 | 2.9 |
| 8-Bromo-cAMP | 1.6E-05 | 4 | **-0.75** | 0.69 | **-0.08** | 0.19 | 9.8 |
| 8-Bromo-cGMP | 1.6E-05 | 4 | **1.22** | 0.43 | **0.48** | 0.51 | 2.5 |
| 8-epi-Prostaglandin F2a | 1.3E-06 | 4 | **-1** | 1.03 | **0.59** | 0.5 | -1.7 |
| 8-methoxymethyl-IBMX | 2.5E-05 | 4 | **-1.26** | 1.81 | **-0.07** | 0.6 | 18.6 |
| 9(S)-HODE | 1.3E-07 | 4 | **-1.66** | 2.37 | **1.53** | 0.85 | -1.1 |
| 9(S)-HPODE | 1.3E-07 | 4 | **-3.56** | 0.06 | **0.55** | 0.39 | -6.4 |
| 9,10-Octadecenoamide | 1.3E-06 | 4 | **-1.34** | 0.56 | **1.64** | 0.35 | -0.8 |
| 9a,11b-Prostaglandin F2 | 1.3E-06 | 4 | **-3.36** | 0.24 | **-0.6** | 0.85 | 5.6 |
| 9-cis Retinoic acid | 1.3E-06 | 4 | **2.56** | 0.53 | **0.38** | 0.97 | 6.8 |
| A-3 | 2.3E-05 | 4 | **19.45** | 1.43 | **0.58** | 0.39 | 33.5 |
| AA-861 | 2.0E-05 | 4 | **17.67** | 1.45 | **-0.03** | 0.13 | -607.7 |
| ABC294640 | 1.8E-05 | 4 | **0.5** | 1.51 | **0.97** | 0.95 | 0.5 |
| Acetyl (N)-s-farnesyl-l-cysteine | 1.8E-05 | 4 | **-0.57** | 1.15 | **-0.35** | 0.47 | 1.7 |
| Ac-Leu-Leu-Nle-CHO | 1.7E-05 | 4 | **28.5** | 3.36 | **21.45** | 1.05 | 1.3 |
| Aconitine | 1.0E-05 | 4 | **5.67** | 0.83 | **1.07** | 0.1 | 5.3 |
| Actinomycin D | 1.4E-05 | 4 | **5.79** | 0.49 | **8.18** | 0.48 | 0.7 |
| Adrenic acid (22:4, n-6) | 1.3E-06 | 4 | **-2.1** | 1.03 | **1.35** | 1.66 | -1.6 |
| AG-1296 | 2.5E-05 | 4 | **0.13** | 1.58 | **0.21** | 0.9 | 0.6 |
| AG1478 | 1.9E-05 | 4 | **5.76** | 0.47 | **3.99** | 1.02 | 1.4 |
| AG213 (Tyrphostin 47) | 3.0E-05 | 4 | **-0.59** | 1.14 | **0.29** | 0.83 | -2.0 |
| AG-370 | 2.6E-05 | 4 | **0.47** | 0.43 | **0.59** | 0.57 | 0.8 |
| AG-490 | 2.3E-05 | 4 | **-0.07** | 0.58 | **1.49** | 0.32 | 0.0 |
| AG-879 | 2.1E-05 | 4 | **-2.84** | 0.64 | **2.32** | 1.29 | -1.2 |
| Ala-Ala-Phe-CMK | 2.0E-05 | 4 | **0.88** | 0.74 | **0.59** | 1.16 | 1.5 |
| Alamethicin | 3.4E-06 | 4 | **2.61** | 2.75 | **1.09** | 0.12 | 2.4 |
| All trans retinoic acid | 1.3E-06 | 4 | **3.65** | 1.82 | **0.86** | 0.67 | 4.3 |
| Alrestatin | 2.6E-05 | 4 | **-1.12** | 0.67 | **-2.08** | 0.3 | 0.5 |
| AM 92016-HCl | 1.4E-05 | 4 | **46.62** | 2.23 | **2.26** | 1.08 | 20.6 |
| AM-251 | 1.3E-06 | 4 | **-1.68** | 1.57 | **1.9** | 0.4 | -0.9 |
| AM-580 | 1.3E-06 | 4 | **0.52** | 0.18 | **1.25** | 0.91 | 0.4 |
| Amantadine?HCl | 3.6E-05 | 4 | **4.42** | 1.41 | **0.5** | 0.08 | 8.8 |
| Amiloride-HCl | 2.5E-05 | 4 | **6.19** | 1.63 | **1.77** | 0.7 | 3.5 |
| Amiodarone?HCl | 9.8E-06 | 4 | **34.91** | 1.72 | **4.49** | 0.15 | 7.8 |
| Anandamide (18:2,n-6) | 1.3E-06 | 4 | **2.61** | 1.09 | **-0.17** | 0.78 | -14.9 |
| Anandamide (20:3,n-6) | 1.3E-06 | 4 | **-3.34** | 0.21 | **-0.91** | 0.07 | 3.7 |
| Anandamide (20:4, n-6) | 1.3E-06 | 4 | **0.42** | 0.54 | **0.27** | 0.54 | 1.6 |
| Anandamide (22:4,n-6) | 1.3E-06 | 4 | **-0.06** | 2.87 | **0.98** | 1.3 | -0.1 |
| Anisomycin | 2.5E-05 | 4 | **12.68** | 2.07 | **11.94** | 0.94 | 1.1 |
| Aphidicolin | 2.0E-05 | 4 | **20.16** | 0.37 | **27.47** | 5.81 | 0.7 |
| Arachidonamide | 1.3E-06 | 4 | **-1.92** | 0.06 | **0.9** | 1.05 | -2.1 |
| Arachidonic acid (20:4, n-6) | 1.3E-06 | 4 | **0.49** | 1.51 | **0.32** | 0.96 | 1.5 |
| Arachidonoyl-PAF | 1.3E-06 | 4 | **1.83** | 1.08 | **1.5** | 1.18 | 1.2 |
| Aristolochic acid | 2.0E-05 | 4 | **-0.15** | 2.53 | **-1.15** | 0.18 | 0.1 |
| Arvanil | 1.5E-05 | 4 | **-0.42** | 0.41 | **-0.01** | 0.26 | 34.6 |
| Ascomycin (FK-520) | 8.4E-06 | 4 | **0.71** | 0.76 | **0.09** | 0.46 | 7.8 |
| B581 | 1.4E-05 | 4 | **-2.34** | 0.46 | **0.52** | 0.17 | -4.5 |
| BADGE | 2.0E-05 | 4 | **98.08** | 3.69 | **101.76** | 1.08 | 1.0 |
| Bafilomycin A1 | 1.1E-06 | 4 | **2.01** | 1.88 | **0.72** | 0.84 | 2.8 |
| BAPTA-AM | 8.7E-06 | 4 | **94.11** | 5.54 | **93.91** | 2.17 | 1.0 |
| BAY 11-7082 | 3.2E-05 | 2 | **131.81** | 2.5 | **154.2** | 1.3 | 0.9 |
| Benzamil-HCl | 1.9E-05 | 4 | **2** | 0.74 | **5.6** | 0.65 | 0.4 |
| Beta-lapachone | 2.8E-05 | 3 | **75.6** | 9.22 | **155.01** | 0.27 | 0.5 |
| Betulinic acid | 1.5E-05 | 4 | **1.77** | 0.99 | **-1.31** | 0.33 | -1.4 |
| bezafibrate | 1.8E-05 | 4 | **1.67** | 1.32 | **-0.12** | 0.12 | -14.3 |
| Blank | 1.3E-06 | 4 | **-2.06** | 0.75 | **1.75** | 0.12 | -1.2 |
| Blebbistatin | 2.3E-05 | 4 | **-2.21** | 0.83 | **2.6** | 0.55 | -0.9 |
| BML-190 | 1.3E-06 | 4 | **2.21** | 1.08 | **1.88** | 0.49 | 1.2 |
| Boc-GVV-CHO | 1.9E-05 | 4 | **6.25** | 0.79 | **2.52** | 1.15 | 2.5 |
| Bongkrekic acid | 1.4E-06 | 4 | **-2.11** | 0.96 | **-0.47** | 0.16 | 4.5 |
| Brefeldin A | 2.4E-05 | 4 | **4.52** | 0.52 | **4.28** | 2.66 | 1.1 |
| Bromo-7-nitroindazole | 2.8E-05 | 4 | **1.87** | 1.02 | **0.51** | 0.34 | 3.7 |
| Bumetanide | 1.8E-05 | 4 | **-1.57** | 1.04 | **1.42** | 0.76 | -1.1 |
| BW-B 70C | 2.1E-05 | 4 | **1.45** | 2.22 | **2.86** | 0.06 | 0.5 |
| C16 Ceramide | 1.3E-06 | 4 | **-1.41** | 1.87 | **0.29** | 0.35 | -4.9 |
| C2 Ceramide | 1.3E-06 | 4 | **0.44** | 1.39 | **2.39** | 1.92 | 0.2 |
| C2 Dihydroceramide | 1.3E-06 | 4 | **1.12** | 0.38 | **2.19** | 0.22 | 0.5 |
| C8 Ceramide | 1.3E-06 | 4 | **1.59** | 1.39 | **0.26** | 1.02 | 6.1 |
| C8 Ceramine | 1.3E-06 | 2 | **-4.56** | 0.41 | **-0.96** | 0.77 | 4.8 |
| C8 Dihydroceramide | 1.3E-06 | 4 | **-1.55** | 0.53 | **1.21** | 0.85 | -1.3 |
| CA-074-Me | 1.7E-05 | 4 | **12.14** | 3.34 | **8.83** | 1.27 | 1.4 |
| Calpeptin | 1.8E-05 | 4 | **10.7** | 1.19 | **3.45** | 1.34 | 3.1 |
| Calphostin C | 8.4E-07 | 4 | **-1.8** | 0.64 | **0.39** | 0.3 | -4.6 |
| Calyculin A | 6.6E-07 | 3 | **119.7** | 13.7 | **118.96** | 1.69 | 1.0 |
| Camptothecin | 1.9E-05 | 4 | **117.99** | 4.88 | **144.75** | 1.45 | 0.8 |
| Cantharidin | 3.4E-05 | 3 | **57.06** | 4.19 | **71.22** | 8.68 | 0.8 |
| CAPE | 2.3E-05 | 4 | **7.28** | 1.42 | **5.39** | 0.6 | 1.4 |
| Capsazepine | 1.8E-05 | 2 | **39.78** | 37.4 | **72.64** | 3.88 | 0.6 |
| Carbacyclin | 1.3E-06 | 4 | **1.49** | 1.48 | **3.56** | 0.77 | 0.4 |
| Castanospermine | 3.5E-05 | 4 | **-1.51** | 1.5 | **1.32** | 0.67 | -1.2 |
| CDC | 2.1E-05 | 4 | **7.36** | 0.52 | **0.9** | 0.93 | 8.2 |
| Cerulenin | 3.0E-05 | 4 | **12.7** | 3.49 | **31.96** | 2.68 | 0.4 |
| CGP-37157 | 2.1E-05 | 4 | **0.7** | 0.44 | **1.22** | 0.65 | 0.6 |
| Chelerythrine | 1.9E-05 | 4 | **13.27** | 3.37 | **13.04** | 2.15 | 1.0 |
| Ciglitazone | 1.3E-06 | 4 | **2.52** | 1.45 | **1.9** | 0.28 | 1.3 |
| Cimaterol | 3.0E-05 | 4 | **2.58** | 2.44 | **2.25** | 0.69 | 1.1 |
| CinnGEL 2Me | 1.3E-05 | 4 | **0.23** | 1.67 | **1.32** | 0.94 | 0.2 |
| Cirazoline | 3.1E-05 | 4 | **0.13** | 0.45 | **3.03** | 0.52 | 0.0 |
| CITCO | 1.5E-05 | 4 | **23.37** | 9.53 | **21.78** | 15.1 | 1.1 |
| Clofibrate | 1.3E-06 | 4 | **2.54** | 1.23 | **2.34** | 0.83 | 1.1 |
| Clonidine | 2.9E-05 | 4 | **3.68** | 1.53 | **2.66** | 0.89 | 1.4 |
| Cloprostenol | 1.3E-06 | 4 | **0.64** | 3.11 | **2.59** | 1.17 | 0.3 |
| Clozapine | 2.0E-05 | 4 | **49.09** | 3.42 | **4.56** | 0.84 | 10.8 |
| C-PAF | 1.3E-06 | 4 | **-3.37** | 0.81 | **0.54** | 0.89 | -6.2 |
| Cyclo [Arg-Gly-Asp-D-Phe-Val] | 1.2E-05 | 4 | **0.26** | 3.07 | **-0.21** | 0.33 | -1.3 |
| Cycloheximide | 2.4E-05 | 4 | **6.97** | 1.42 | **14.5** | 3.28 | 0.5 |
| Cycloheximide-N-ethylethanoate | 1.8E-05 | 4 | **1.05** | 0.9 | **1.26** | 1.03 | 0.8 |
| Cyclopamine | 1.6E-05 | 4 | **10.68** | 5.04 | **-0.29** | 0.59 | -37.2 |
| Cyclopiazonic acid | 2.0E-05 | 4 | **-6.61** | 0.31 | **-4.26** | 0.81 | 1.6 |
| Cyclosporin A | 5.5E-06 | 4 | **2.31** | 2.05 | **-0.21** | 0.86 | -11.3 |
| Cypermethrin | 1.6E-05 | 4 | **5.4** | 1.04 | **1.79** | 1.24 | 3.0 |
| Cytochalasin B | 1.4E-05 | 4 | **202.79** | 3.96 | **21.52** | 0.79 | 9.4 |
| Cytochalasin D | 1.3E-05 | 4 | **36.09** | 4.21 | **0.68** | 0.39 | 53.0 |
| D12-Prostaglandin J2 | 1.3E-06 | 3 | **-0.04** | 0.79 | **-1** | 0.66 | 0.0 |
| D609 | 2.5E-05 | 3 | **-1.67** | 2.03 | **-1.01** | 0.69 | 1.7 |
| Damnacanthal | 2.4E-05 | 4 | **-2.36** | 1.47 | **0.08** | 0.29 | -28.7 |
| Dantrolene | 2.1E-05 | 4 | **-2.17** | 0.95 | **-1.16** | 0.66 | 1.9 |
| Decoyinine | 2.4E-05 | 4 | **-1.94** | 1.02 | **0.49** | 0.23 | -4.0 |
| Decylubiquinone | 2.1E-05 | 4 | **1.77** | 1.1 | **1** | 0.87 | 1.8 |
| Deprenyl | 3.6E-05 | 4 | **1.31** | 1.54 | **0.2** | 1.24 | 6.6 |
| D-erythro-MAPP | 1.3E-06 | 4 | **-2.84** | 1.17 | **-0.45** | 0.41 | 6.3 |
| Dexamethasone | 1.7E-05 | 4 | **1.91** | 2.35 | **-2.55** | 0.5 | -0.8 |
| Diazoxide | 2.9E-05 | 4 | **-3.22** | 1.01 | **-0.49** | 0.77 | 6.6 |
| Dibutyrylcyclic AMP | 1.4E-05 | 4 | **-1.99** | 0.98 | **-0.06** | 1.22 | 31.4 |
| Dibutyrylcyclic GMP | 1.4E-05 | 4 | **0.24** | 1.74 | **1.05** | 0.32 | 0.2 |
| Dichlorobenzamil-HCl | 1.6E-05 | 4 | **2.17** | 1.06 | **4.7** | 2.86 | 0.5 |
| Dihomo-gamma-linolenic acid | 1.3E-06 | 4 | **-2.56** | 0.57 | **-0.79** | 0.23 | 3.2 |
| Dihydrosphingosine | 1.3E-06 | 4 | **-0.97** | 1.98 | **0.25** | 0.6 | -3.9 |
| Diidolylmethane | 1.3E-06 | 4 | **1.6** | 1.78 | **0.13** | 0.17 | 12.2 |
| Diltiazem-HCl | 1.5E-05 | 4 | **33.28** | 0.83 | **2.47** | 0.16 | 13.5 |
| Dimethyloxaloylglycine | 1.3E-06 | 4 | **-3.01** | 0.81 | **0.43** | 0.4 | -7.1 |
| Dipalmitoylphosphatidic acid | 1.3E-06 | 4 | **-1.87** | 1.62 | **-0.03** | 0.26 | 55.0 |
| Diphenyleneiodonium | 2.4E-05 | 4 | **67.93** | 5.81 | **52.09** | 9.73 | 1.3 |
| Dipyridamole | 1.3E-05 | 4 | **10.02** | 2.79 | **4.35** | 1.98 | 2.3 |
| DL-Dihydrosphingosine | 1.3E-06 | 4 | **-0.92** | 0.39 | **-0.07** | 0.17 | 13.9 |
| DL-PDMP | 1.3E-06 | 4 | **-2.31** | 0.32 | **-0.25** | 0.37 | 9.2 |
| DL-PPMP | 1.3E-06 | 4 | **-1.07** | 0.5 | **0.65** | 0.65 | -1.7 |
| Docosahexaenoic acid(22:6 n-3) | 1.3E-06 | 4 | **-2.15** | 0.85 | **-0.69** | 0.4 | 3.1 |
| Docosapentaenoic acid | 1.3E-06 | 4 | **-1.04** | 0.92 | **-1.02** | 0.45 | 1.0 |
| Docosatrienoic acid (22:3 n-3) | 1.3E-06 | 4 | **-2.77** | 0.93 | **-0.74** | 0.47 | 3.8 |
| Doxorubicin | 1.2E-05 | 3 | **-8.22** | 0.3 | **-3.14** | 0.42 | 2.6 |
| DRB (Benzimidazole) | 2.1E-05 | 4 | **4.44** | 2.07 | **7.62** | 1.98 | 0.6 |
| E-4031 | 1.7E-05 | 4 | **-1.01** | 0.91 | **-0.31** | 0.66 | 3.2 |
| E6 berbamine | 8.8E-06 | 4 | **110.58** | 0.46 | **33.56** | 1.73 | 3.3 |
| E-64-d | 2.0E-05 | 3 | **9.26** | 9.24 | **2.88** | 0.85 | 3.2 |
| Ebselen | 2.4E-05 | 4 | **-0.44** | 0.37 | **-0.07** | 0.41 | 6.4 |
| E-Capsaicin | 2.2E-05 | 4 | **1.19** | 1.29 | **1.69** | 0.69 | 0.7 |
| Eicosa-5,8-dienoic acid (20:2 n-12) | 1.3E-06 | 4 | **-0.14** | 1.02 | **-1.44** | 0.19 | 0.1 |
| Eicosadienoic acid (20:2 n-6) | 1.3E-06 | 4 | **-2.19** | 0.72 | **0.45** | 0.65 | -4.9 |
| Eicosapentaenoic acid (20:5 n-3) | 1.3E-06 | 4 | **-2.46** | 0.47 | **-0.29** | 0.44 | 8.4 |
| Eicosatrienoic acid (20:3 n-3) | 1.3E-06 | 4 | **-1.76** | 0.96 | **0.76** | 0.18 | -2.3 |
| Enantio-PAF C16 | 1.3E-06 | 4 | **-1.42** | 0.86 | **0.97** | 1.59 | -1.5 |
| Estradiol | 2.5E-05 | 4 | **5.64** | 1.79 | **14.64** | 2.61 | 0.4 |
| Etoposide | 1.1E-05 | 4 | **2.87** | 0.91 | **16.93** | 4.56 | 0.2 |
| Farnesylthioacetic acid | 1.3E-06 | 4 | **-1.73** | 1.16 | **0.68** | 0.66 | -2.6 |
| FCCP | 2.6E-05 | 4 | **5.68** | 2.79 | **16.24** | 0.08 | 0.4 |
| Fipronil | 1.5E-05 | 4 | **-2.97** | 1.16 | **-1.37** | 0.05 | 2.2 |
| FK-506 | 8.3E-06 | 4 | **-0.84** | 0.36 | **1.37** | 1.74 | -0.6 |
| Flecainide acetate | 1.4E-05 | 4 | **0.34** | 0.52 | **1.38** | 0.38 | 0.2 |
| Flufenamic acid | 2.4E-05 | 4 | **-2.07** | 0.37 | **-0.67** | 0.07 | 3.1 |
| Flunarizine-2HCl | 1.4E-05 | 4 | **-0.95** | 0.45 | **-0.33** | 0.52 | 2.9 |
| Fluprostenol | 1.3E-06 | 4 | **-2.96** | 0.89 | **0.7** | 0.41 | -4.2 |
| Fluspirilene | 1.4E-05 | 4 | **12.66** | 1.71 | **1.35** | 0.41 | 9.4 |
| Forskolin | 1.6E-05 | 4 | **10.52** | 0.55 | **8.03** | 0.6 | 1.3 |
| FPL-64176 | 1.9E-05 | 4 | **-0.2** | 1.81 | **-0.23** | 0.61 | 0.9 |
| Fpp2 | 2.3E-05 | 1 | **-6.55** | - | **-0.53** | - | 12.3 |
| Fumonisin B1 | 9.2E-06 | 4 | **0** | 0.09 | **0.58** | 0.26 | 0.0 |
| Furoxan | 3.6E-05 | 4 | **52.32** | 4.12 | **84.85** | 10.2 | 0.6 |
| Gamma-linolenic acid (18:3 n-6) | 1.3E-06 | 4 | **0.09** | 0.21 | **-0.37** | 0.71 | -0.2 |
| Geldanamycin | 1.2E-05 | 4 | **49.82** | 3.68 | **61.51** | 4.08 | 0.8 |
| Genistein | 2.6E-05 | 4 | **4.56** | 1.23 | **8.92** | 3.08 | 0.5 |
| GF-109203X | 1.6E-05 | 3 | **95.71** | 1.03 | **14.68** | 1.79 | 6.5 |
| Gliotoxin | 2.0E-05 | 4 | **130.88** | 1.29 | **151.16** | 1.98 | 0.9 |
| Glipizide | 1.5E-05 | 4 | **-1.54** | 1.38 | **-1.89** | 0.22 | 0.8 |
| Glyburide | 1.4E-05 | 4 | **-2.15** | 0.52 | **-0.78** | 0.65 | 2.8 |
| GM6001 | 1.7E-05 | 4 | **0.38** | 1.12 | **0.81** | 0.2 | 0.5 |
| Go6976 | 1.8E-05 | 3 | **-3.86** | 2.33 | **22.66** | 11.9 | -0.2 |
| Grayanotoxin III | 1.8E-05 | 4 | **-2.44** | 0.62 | **-0.44** | 0.27 | 5.6 |
| GW-5074 | 1.3E-05 | 4 | **8.58** | 1.95 | **1.31** | 0.84 | 6.6 |
| GW-9662 | 2.4E-05 | 4 | **8.9** | 1.85 | **21.64** | 1.09 | 0.4 |
| H7 | 2.3E-05 | 4 | **-1.92** | 0.59 | **6.84** | 3.07 | -0.3 |
| H-89 | 1.5E-05 | 4 | **35.12** | 5.41 | **4.35** | 1.9 | 8.1 |
| H9 | 2.1E-05 | 4 | **-1.71** | 1.87 | **0.01** | 0.55 | -136.3 |
| HA-1004 | 2.3E-05 | 4 | **-3.74** | 1.57 | **-0.48** | 0.14 | 7.8 |
| HA1077 | 1.8E-05 | 4 | **-3.75** | 0.63 | **-1.01** | 0.09 | 3.7 |
| HBDDE | 2.0E-05 | 4 | **-3.99** | 1 | **-2.19** | 0.2 | 1.8 |
| Hinokitiol | 4.1E-05 | 4 | **-2.81** | 1.44 | **8.85** | 2.67 | -0.3 |
| Histamine | 6.0E-05 | 4 | **-1.59** | 0.34 | **-1.75** | 0.56 | 0.9 |
| HNMPA-(AM)3 | 1.5E-05 | 4 | **-3.71** | 0.67 | **3.18** | 1.38 | -1.2 |
| IB-MECA | 1.3E-05 | 4 | **1.92** | 1.47 | **0.94** | 0.47 | 2.0 |
| IBMX | 3.0E-05 | 4 | **0.32** | 0.53 | **-0.88** | 0.91 | -0.4 |
| ICRF-193 | 2.4E-05 | 3 | **24.84** | 7.55 | **75.78** | 1.9 | 0.3 |
| Ikarugamycin | 1.4E-05 | 4 | **130.11** | 1.76 | **137.45** | 7.53 | 1.0 |
| Indirubin | 2.5E-05 | 4 | **5.56** | 2.08 | **2.4** | 0.69 | 2.3 |
| Indirubin-3'-monoxime | 2.4E-05 | 4 | **3.23** | 0.82 | **6.63** | 2.99 | 0.5 |
| Indomethacin | 1.9E-05 | 4 | **48.11** | 10.8 | **6.26** | 2.31 | 7.7 |
| Ionomycin | 9.4E-06 | 4 | **0.62** | 3.69 | **-0.36** | 0.51 | -1.7 |
| Juglone | 3.8E-05 | 4 | **-8.44** | 0.72 | **-4.96** | 0.53 | 1.7 |
| K252A | 1.4E-06 | 4 | **8.74** | 2.91 | **13.39** | 7.51 | 0.7 |
| Kavain (+/-) | 2.9E-05 | 4 | **-1.63** | 0.79 | **0.27** | 0.4 | -6.0 |
| KN-62 | 9.2E-06 | 4 | **-0.24** | 1.26 | **0.91** | 0.58 | -0.3 |
| KT-5720 | 1.2E-06 | 4 | **2.64** | 1.6 | **-1.17** | 0.13 | -2.3 |
| L-744,832 | 1.1E-05 | 4 | **-0.2** | 1.59 | **5.77** | 0.59 | 0.0 |
| Latrunculin B | 1.7E-05 | 4 | **57.38** | 1.69 | **15.91** | 0.88 | 3.6 |
| Lavendustin A | 1.8E-05 | 4 | **4.95** | 1.03 | **-0.1** | 1.12 | -48.2 |
| L-cis-Diltiazem?HCl | 1.5E-05 | 4 | **-1.77** | 0.93 | **0.14** | 0.3 | -12.3 |
| L-erythro-MAPP | 1.3E-06 | 4 | **-1.04** | 0.62 | **0.06** | 0.76 | -16.0 |
| Leukotoxin A (9,10-EODE) | 1.3E-07 | 4 | **-0.26** | 1.24 | **0.37** | 0.76 | -0.7 |
| Leukotoxin B (12,13-EODE) | 1.3E-07 | 4 | **-1.85** | 0.64 | **-1.09** | 0.49 | 1.7 |
| Leukotriene B4 | 1.3E-07 | 4 | **-2.16** | 0.29 | **0.06** | 0.27 | -37.2 |
| Leukotriene C4 | 1.3E-07 | 4 | **-2.44** | 1.02 | **-0.46** | 1.08 | 5.3 |
| Leukotriene D4 | 1.3E-07 | 4 | **-0.58** | 0.2 | **-0.58** | 0.21 | 1.0 |
| Leukotriene E4 | 1.3E-07 | 4 | **-2.84** | 0.33 | **0.22** | 0.18 | -12.7 |
| Leupeptin | 1.6E-05 | 4 | **54.73** | 24.6 | **108.71** | 7.25 | 0.5 |
| LFM-A13 | 1.9E-05 | 4 | **-0.79** | 0.79 | **2.23** | 0.87 | -0.4 |
| Lidocaine-HCl-H2O | 2.3E-05 | 4 | **1.82** | 0.82 | **-0.83** | 0.27 | -2.2 |
| Linoleamide | 1.3E-06 | 4 | **-2.01** | 0.42 | **-0.36** | 0.4 | 5.5 |
| Linoleic acid | 1.3E-06 | 4 | **-2.69** | 0.89 | **0.04** | 0.91 | -70.8 |
| Linolenic acid (18:3 n-3) | 1.3E-06 | 4 | **-0.58** | 0.68 | **1.15** | 1.03 | -0.5 |
| L-NAME | 2.9E-05 | 4 | **-0.63** | 0.25 | **-0.52** | 1.1 | 1.2 |
| L-NASPA | 1.3E-06 | 4 | **-1.52** | 0.32 | **-0.41** | 0.31 | 3.7 |
| Loperamide-HCl | 1.3E-05 | 4 | **15.53** | 2.08 | **1.27** | 0.95 | 12.2 |
| LY-171883 | 1.3E-06 | 4 | **-1.22** | 0.96 | **-0.95** | 0.16 | 1.3 |
| LY-294002 | 2.2E-05 | 4 | **15.04** | 1.11 | **66.28** | 7.73 | 0.2 |
| LY-83583 | 2.7E-05 | 4 | **133.77** | 0.31 | **157.39** | 0.84 | 0.9 |
| Lycorine | 2.3E-05 | 3 | **0.95** | 1.88 | **-0.39** | 0.38 | -2.4 |
| Lyso-PAF C16 | 1.3E-06 | 4 | **-1.41** | 0.5 | **-0.42** | 0.31 | 3.4 |
| Lysophosphatidic acid-Na | 1.3E-06 | 4 | **-2.3** | 1.31 | **-0.39** | 0.47 | 6.0 |
| Manoalide | 1.6E-05 | 4 | **4.85** | 4.27 | **6.12** | 0.38 | 0.8 |
| Manumycin A | 1.2E-05 | 4 | **15.06** | 3.5 | **6.27** | 2.44 | 2.4 |
| Mastoparan | 4.0E-06 | 4 | **3.16** | 1.45 | **5.87** | 0.36 | 0.5 |
| MBCQ | 2.1E-05 | 4 | **0.07** | 1.05 | **2.75** | 1 | 0.0 |
| MDL-28170 | 1.7E-05 | 4 | **11.04** | 1.98 | **1.58** | 0.48 | 7.0 |
| Mead acid (20:3 n-9) | 1.3E-06 | 4 | **-2.34** | 0.81 | **-0.16** | 0.48 | 14.2 |
| Mead ethanolamide | 1.3E-06 | 4 | **-3.53** | 0.23 | **-0.47** | 0.34 | 7.5 |
| Methoprene acid | 1.3E-06 | 4 | **-1.22** | 0.72 | **-0.11** | 0.51 | 11.1 |
| Methotrexate | 1.5E-05 | 4 | **-2.78** | 0.04 | **10.75** | 2.14 | -0.3 |
| Methoxyverapimil-HCl | 1.3E-05 | 4 | **-2.01** | 0.2 | **-0.8** | 0.15 | 2.5 |
| Mevinolin (Lovastatin) | 1.7E-05 | 4 | **101.94** | 1.36 | **76.02** | 7.08 | 1.3 |
| MG-132 | 1.4E-05 | 4 | **-0.94** | 1.29 | **7.59** | 0.5 | -0.1 |
| Milrinone | 3.2E-05 | 4 | **-3.76** | 0.58 | **-0.66** | 0.04 | 5.7 |
| Minoxidil | 2.3E-05 | 3 | **-0.88** | 0.56 | **-1.77** | 0.51 | 0.5 |
| Minoxidil sulfate | 3.2E-05 | 4 | **-1.42** | 0.71 | **0.02** | 1.07 | -77.4 |
| Misoprostol, free acid | 1.3E-06 | 4 | **-1.94** | 0.26 | **0.14** | 0.65 | -13.9 |
| Mitomycin C | 2.0E-05 | 3 | **115.26** | 3.51 | **97.13** | 1.76 | 1.2 |
| ML7 | 1.6E-05 | 4 | **82.72** | 5.64 | **56.01** | 4.16 | 1.5 |
| ML9 | 2.1E-05 | 4 | **92.51** | 2.5 | **49.36** | 4.05 | 1.9 |
| MnTBAP chloride | 7.6E-06 | 4 | **-2.17** | 1.17 | **-0.25** | 0.2 | 8.6 |
| Monastrol | 2.3E-05 | 4 | **5.54** | 0.57 | **6.65** | 2.9 | 0.8 |
| Monensin sodium | 9.6E-06 | 4 | **27.11** | 7.36 | **4.2** | 1.83 | 6.5 |
| MY-5445 | 2.0E-05 | 4 | **1.5** | 2.09 | **-0.17** | 1.46 | -8.7 |
| Mycophenolic acid | 2.1E-05 | 4 | **22.77** | 6.64 | **60.36** | 6.41 | 0.4 |
| N,N-Dimethylsphingosine | 1.3E-06 | 4 | **11.74** | 1.11 | **1.13** | 1.02 | 10.4 |
| N9-Isopropylolomoucine | 2.0E-05 | 4 | **8.47** | 2.75 | **15.53** | 3.13 | 0.6 |
| N-Acetyl-leukotriene E4 | 1.3E-07 | 4 | **-2.74** | 0.38 | **-0.12** | 0.55 | 23.3 |
| N-acetyl-S-geranylgeranyl-L-Cysteine | 1.3E-06 | 4 | **-0.61** | 1.67 | **-0.45** | 0.76 | 1.4 |
| N-Acetyl-S-geranyl-L-cysteine | 1.3E-06 | 4 | **-1.73** | 0.07 | **0.04** | 0.33 | -39.6 |
| Nafamostat mesylate | 1.5E-05 | 4 | **30.66** | 8.22 | **46.32** | 4.34 | 0.7 |
| NapSul-Ile-Trp-CHO | 1.4E-05 | 4 | **0.76** | 0.64 | **-0.1** | 1.09 | -7.9 |
| N-arachidonoylglycine | 1.3E-06 | 4 | **-1.93** | 1.34 | **-1.29** | 0.16 | 1.5 |
| Nicardipine-HCl | 1.3E-05 | 4 | **0.88** | 0.34 | **0.55** | 0.21 | 1.6 |
| Nifedipine | 1.9E-05 | 4 | **1.08** | 1.21 | **0.29** | 1.22 | 3.8 |
| Niflumic acid | 2.4E-05 | 4 | **0.92** | 2.32 | **-0.06** | 0.43 | -15.5 |
| Nigericin | 9.2E-06 | 4 | **19.27** | 2.4 | **20.13** | 2.13 | 1.0 |
| Niguldipine | 1.0E-05 | 4 | **27.84** | 1.57 | **0.67** | 0.96 | 41.4 |
| Nimesulide | 2.2E-05 | 4 | **4.8** | 5.79 | **6.55** | 6.76 | 0.7 |
| Nimodipine | 1.6E-05 | 4 | **0.63** | 0.16 | **0.43** | 0.31 | 1.5 |
| Nitrendipine | 1.9E-05 | 4 | **1.13** | 1.54 | **-0.69** | 0.79 | -1.6 |
| N-linoleoylglycine | 1.3E-06 | 4 | **-3.57** | 0.58 | **-0.4** | 0.28 | 8.9 |
| Nocodazole | 2.2E-05 | 4 | **26.36** | 5.45 | **17.26** | 12.6 | 1.5 |
| N-Phenylanthranilic acid | 3.1E-05 | 4 | **0.49** | 2.2 | **-2.08** | 0.86 | -0.2 |
| NPPB | 2.2E-05 | 4 | **-2.1** | 1.29 | **-2.72** | 0.75 | 0.8 |
| NS-1619 | 1.8E-05 | 4 | **-4.53** | 0.47 | **-1.96** | 0.71 | 2.3 |
| NS-398 | 2.1E-05 | 4 | **5.12** | 2.07 | **1.06** | 0.92 | 4.8 |
| NSC-95397 | 2.2E-05 | 4 | **52.82** | 4.38 | **50.5** | 6.46 | 1.1 |
| Okadaic acid | 8.3E-07 | 4 | **14.86** | 2.49 | **18.9** | 1.7 | 0.8 |
| Oligomycin A | 8.4E-06 | 4 | **26.34** | 10.8 | **14.02** | 9.23 | 1.9 |
| Olomoucine | 2.2E-05 | 4 | **-0.97** | 1.32 | **-0.5** | 0.68 | 1.9 |
| Ouabain | 1.1E-05 | 4 | **44.55** | 5.27 | **37.81** | 7.53 | 1.2 |
| PAF C16 | 1.3E-06 | 4 | **-2.29** | 1.1 | **0.03** | 0.97 | -83.4 |
| PAF C18 | 1.3E-06 | 4 | **-0.75** | 0.86 | **0.19** | 0.24 | -3.9 |
| PAF C18:1 | 1.3E-06 | 4 | **-1.1** | 0.18 | **-0.13** | 0.65 | 8.8 |
| Palmitylethanolamide | 1.3E-06 | 4 | **-0.68** | 0.89 | **-0.47** | 0.48 | 1.4 |
| Parthenolide | 2.7E-05 | 2 | **124** | 1.51 | **113.57** | 9.92 | 1.1 |
| Paxilline | 1.5E-05 | 4 | **-2.84** | 0.23 | **-0.09** | 0.59 | 30.6 |
| PCA 4248 | 1.8E-05 | 4 | **5.12** | 2.18 | **4.5** | 1.88 | 1.1 |
| PCO-400 | 2.2E-05 | 4 | **-2.07** | 0.36 | **-0.33** | 0.84 | 6.3 |
| PD 98059 | 2.5E-05 | 4 | **26.64** | 4.57 | **37** | 11.6 | 0.7 |
| Penitrem A | 1.1E-05 | 4 | **-0.52** | 0.46 | **-2.18** | 0.16 | 0.2 |
| Pepstatin | 9.7E-06 | 4 | **2.53** | 3.91 | **-1.63** | 0.98 | -1.6 |
| Phenamil | 2.2E-05 | 4 | **4.34** | 2.39 | **-0.02** | 1.2 | -202.4 |
| Phenoxybenzamine | 2.2E-05 | 4 | **5.5** | 1.79 | **1.39** | 0.61 | 4.0 |
| Phentolamine | 2.4E-05 | 4 | **-3.38** | 0.39 | **-0.38** | 0.54 | 8.9 |
| Phenytoin | 2.6E-05 | 4 | **-2.35** | 0.68 | **-0.71** | 0.59 | 3.3 |
| Phorbol 12-myristate 13-acetate | 1.1E-05 | 4 | **22.54** | 10.2 | **0.89** | 1.02 | 25.2 |
| Piceatannol | 2.7E-05 | 4 | **3.99** | 1.06 | **6.53** | 1.5 | 0.6 |
| Pifithrin-α | 2.3E-05 | 4 | **5.38** | 3.42 | **7.48** | 3.46 | 0.7 |
| Pimozide | 1.4E-05 | 4 | **-2.09** | 1.66 | **-1.2** | 0.23 | 1.7 |
| Pinacidil | 2.7E-05 | 4 | **-3.92** | 0.64 | **-0.64** | 0.63 | 6.2 |
| Piroxicam | 2.0E-05 | 4 | **-2.06** | 0.78 | **-2.36** | 0.34 | 0.9 |
| PP1 | 2.4E-05 | 4 | **-1.67** | 1.09 | **-2.86** | 0.93 | 0.6 |
| PP2 | 2.2E-05 | 4 | **-0.61** | 0.38 | **-0.77** | 0.34 | 0.8 |
| Prazocin | 1.7E-05 | 4 | **62.23** | 3.85 | **67.19** | 3.7 | 0.9 |
| Pregnenolone-16α-carbonitrile | 2.0E-05 | 4 | **27.84** | 7.95 | **3.48** | 2.65 | 8.0 |
| PRIMA-1 | 3.6E-05 | 4 | **-2.14** | 1.37 | **-0.89** | 0.36 | 2.4 |
| Procainamide | 2.8E-05 | 4 | **-1.54** | 0.74 | **-1.54** | 0.71 | 1.0 |
| Propafenone | 2.0E-05 | 4 | **34.62** | 0.85 | **-0.73** | 0.65 | -47.7 |
| Propidium iodide | 1.0E-05 | 3 | **-8.71** | 0.22 | **-6.19** | 0.08 | 1.4 |
| Prostaglandin D2 | 1.3E-06 | 4 | **0.28** | 0.75 | **0.44** | 0.86 | 0.6 |
| Prostaglandin E1 | 1.3E-06 | 4 | **-0.5** | 1.12 | **0.82** | 0.88 | -0.6 |
| Prostaglandin E2 | 1.3E-06 | 4 | **-1.1** | 1.18 | **0.03** | 0.46 | -36.2 |
| Prostaglandin F1a | 1.3E-06 | 4 | **-0.37** | 1.14 | **0.43** | 0.93 | -0.9 |
| Prostaglandin A1 | 1.3E-06 | 4 | **-2.08** | 1.68 | **-0.12** | 0.26 | 18.0 |
| Prostaglandin A2 | 1.3E-06 | 4 | **-0.89** | 1.41 | **0.61** | 0.41 | -1.5 |
| Prostaglandin B1 | 1.3E-06 | 4 | **-0.74** | 0.98 | **-1.16** | 0.18 | 0.6 |
| Prostaglandin B2 | 1.3E-06 | 4 | **-1.74** | 0.15 | **0.52** | 0.4 | -3.3 |
| Prostaglandin F2a | 1.3E-06 | 4 | **-1.14** | 0.77 | **0.69** | 0.14 | -1.6 |
| Prostaglandin I2 | 1.3E-06 | 4 | **-0.84** | 0.76 | **0.65** | 1.34 | -1.3 |
| Puromycin?2HCl | 1.3E-05 | 4 | **109.8** | 7.72 | **113.01** | 17.5 | 1.0 |
| QNZ | 1.9E-05 | 4 | **-1.5** | 0.08 | **8.98** | 1.52 | -0.2 |
| Quercetin-2H2O | 2.0E-05 | 4 | **11.39** | 1.74 | **5.94** | 2.87 | 1.9 |
| Quinidine-HCl-H2O | 1.8E-05 | 4 | **-0.33** | 0.51 | **0.26** | 0.75 | -1.3 |
| Quinine-HCl-2H2O | 1.7E-05 | 4 | **-1.17** | 0.04 | **0.41** | 0.34 | -2.9 |
| QX-314 | 1.9E-05 | 4 | **-2.01** | 1.04 | **-1.28** | 0.85 | 1.6 |
| R(+)-IAA-94 | 1.9E-05 | 4 | **-1.2** | 0.9 | **1.36** | 0.13 | -0.9 |
| Rapamycin | 7.3E-06 | 4 | **-3.33** | 0.6 | **3.07** | 0.67 | -1.1 |
| Resveratrol | 2.9E-05 | 4 | **4.74** | 4.86 | **-1.38** | 0.36 | -3.5 |
| REV-5901 | 1.3E-06 | 4 | **0.04** | 1.93 | **0.35** | 0.43 | 0.1 |
| RG-14620 | 2.4E-05 | 4 | **2.81** | 1.03 | **3.58** | 2.99 | 0.8 |
| RHC-80267 | 1.7E-05 | 4 | **3.96** | 0.77 | **0.06** | 1.42 | 70.0 |
| RK-682 | 1.8E-05 | 3 | **-0.62** | 1.16 | **-0.63** | 0.85 | 1.0 |
| Ro 20-1724 | 2.4E-05 | 4 | **3.4** | 2.57 | **3.6** | 2.49 | 1.0 |
| Ro 31-8220 | 1.5E-05 | 4 | **55.94** | 7.66 | **27.98** | 3.36 | 2.0 |
| Rolipram | 2.4E-05 | 4 | **5.33** | 1.3 | **2.81** | 0.42 | 1.9 |
| Roscovitine | 1.9E-05 | 4 | **11.05** | 4.86 | **19.71** | 4.75 | 0.6 |
| Rottlerin | 1.3E-05 | 4 | **4.01** | 0.89 | **1.09** | 0.31 | 3.7 |
| RWJ-60475-(AM)3 | 1.1E-05 | 4 | **4.74** | 3.09 | **0.35** | 0.52 | 13.4 |
| Ryanodine | 1.4E-05 | 4 | **-3.79** | 0.83 | **-0.69** | 0.52 | 5.5 |
| SB 202190 | 2.0E-05 | 4 | **-2.79** | 0.51 | **5.64** | 1.94 | -0.5 |
| SB 203580 | 1.8E-05 | 4 | **6.45** | 1.12 | **-1.71** | 0.61 | -3.8 |
| SB-415286 | 1.9E-05 | 4 | **1.44** | 0.49 | **0.58** | 0.96 | 2.5 |
| SB-431542 | 1.7E-05 | 4 | **17.69** | 8.35 | **4.51** | 3.49 | 3.9 |
| SDZ-201106 | 1.4E-05 | 4 | **0.98** | 0.84 | **-0.58** | 0.42 | -1.7 |
| Serotonin | 3.8E-05 | 4 | **4.12** | 1.49 | **0.02** | 0.86 | 219.8 |
| S-farnesyl-L-cysteine | 1.3E-06 | 4 | **1.55** | 0.87 | **-0.05** | 0.4 | -30.0 |
| Shikonin | 2.3E-05 | 4 | **25.06** | 13.9 | **23.19** | 4.57 | 1.1 |
| Siguazodan | 2.3E-05 | 4 | **0.28** | 0.85 | **0.33** | 1.03 | 0.8 |
| SKF-96365 | 1.8E-05 | 4 | **-2.17** | 1.11 | **-0.22** | 0.71 | 9.9 |
| SP-600125 | 3.0E-05 | 4 | **-1.16** | 0.86 | **8.36** | 2.01 | -0.1 |
| Sphingosine | 1.3E-06 | 4 | **1.72** | 1.82 | **-0.18** | 0.46 | -9.4 |
| Splitomycin | 3.4E-05 | 4 | **1.89** | 2.42 | **1.19** | 0.44 | 1.6 |
| SQ22536 | 3.3E-05 | 4 | **1.52** | 2.74 | **-1.8** | 0.42 | -0.8 |
| SQ-29548 | 1.3E-06 | 4 | **0.92** | 0.93 | **0.12** | 0.52 | 7.7 |
| staurosporine | 1.4E-05 | 4 | **94.7** | 0.46 | **93.51** | 1.02 | 1.0 |
| SU-4312 | 2.5E-05 | 4 | **4.66** | 0.45 | **2.61** | 0.09 | 1.8 |
| swainsonine | 3.9E-05 | 4 | **7.08** | 2.48 | **2.93** | 1.23 | 2.4 |
| Tamoxifen | 1.8E-05 | 4 | **85.72** | 4.25 | **7.52** | 3.35 | 11.4 |
| Tanshinone IIA | 2.3E-05 | 4 | **2.82** | 2.05 | **1.72** | 0.93 | 1.6 |
| Taxol = Paclitaxel | 7.8E-06 | 4 | **33.72** | 11.9 | **62.52** | 5.71 | 0.5 |
| Tetrahydrocannabinol-7-oic acid | 1.3E-06 | 4 | **0.45** | 1.42 | **0.29** | 0.55 | 1.5 |
| Tetrandine | 1.1E-05 | 4 | **4.16** | 2.92 | **-1.76** | 0.98 | -2.4 |
| Thapsigargin | 1.0E-05 | 4 | **47.37** | 2.63 | **66.44** | 4.84 | 0.7 |
| Thiocitrulline [L-Thiocitrulline] | 3.5E-05 | 4 | **-2.03** | 0.23 | **-1.88** | 0.83 | 1.1 |
| Thiorphan | 2.6E-05 | 4 | **0.74** | 1.92 | **-0.45** | 0.19 | -1.7 |
| TMB-8 | 1.7E-05 | 4 | **9.81** | 0.96 | **3.76** | 0.36 | 2.6 |
| Tolazamide | 2.1E-05 | 4 | **-2.75** | 0.16 | **-0.21** | 0.76 | 13.1 |
| Tolbutamide | 2.5E-05 | 4 | **-1** | 0.86 | **-0.19** | 0.35 | 5.3 |
| Tosyl-Phe-CMK (TPCK) | 1.9E-05 | 4 | **-0.17** | 0.55 | **1.48** | 0.64 | -0.1 |
| TPEN | 1.6E-05 | 4 | **124.5** | 2.46 | **136.55** | 3.74 | 0.9 |
| Trequinsin | 1.6E-05 | 4 | **0.93** | 0.56 | **5.26** | 0.26 | 0.2 |
| Trichostatin-A | 2.2E-05 | 3 | **76.24** | 10.7 | **88.88** | 6.91 | 0.9 |
| Trifluoperazine | 1.4E-05 | 4 | **114.1** | 3.77 | **83.4** | 3.79 | 1.4 |
| TRIM | 3.1E-05 | 4 | **-1.36** | 0.89 | **0.64** | 0.59 | -2.1 |
| Triptolide | 1.9E-05 | 4 | **23.36** | 5.69 | **26.72** | 4.47 | 0.9 |
| TTNPB | 1.3E-06 | 4 | **-0.52** | 0.42 | **1.63** | 0.51 | -0.3 |
| Tunicamycin | 9.3E-06 | 4 | **119.29** | 4.8 | **85.42** | 4.03 | 1.4 |
| Tyrphostin 1 | 3.6E-05 | 4 | **1.45** | 1.41 | **0.68** | 0.97 | 2.2 |
| Tyrphostin 9 | 2.4E-05 | 4 | **39.26** | 3.6 | **34.43** | 3.86 | 1.1 |
| Tyrphostin AG-126 | 3.1E-05 | 4 | **0.31** | 0.51 | **0.46** | 1.23 | 0.7 |
| Tyrphostin AG-825 | 1.7E-05 | 4 | **2.38** | 1.18 | **-2.5** | 0.09 | -1.0 |
| Tyrphostin-8 | 3.9E-05 | 4 | **-2.34** | 0.72 | **1.62** | 1.24 | -1.5 |
| U-0126 | 1.8E-05 | 4 | **60.45** | 9.13 | **46.52** | 14.9 | 1.3 |
| U-37883A | 1.9E-05 | 4 | **3.58** | 2.04 | **10.98** | 0.95 | 0.3 |
| U-46619 | 1.3E-06 | 3 | **3.67** | 0.04 | **1.08** | 0.29 | 3.4 |
| U-50488 | 1.8E-05 | 4 | **20.89** | 1.93 | **4.69** | 0.89 | 4.5 |
| U73122 | 1.4E-05 | 4 | **74.99** | 4.69 | **23.76** | 0.76 | 3.2 |
| U-74389G | 1.1E-05 | 4 | **4.79** | 1.38 | **2.14** | 1.11 | 2.2 |
| U-75302 | 1.3E-07 | 4 | **2.61** | 1.42 | **1.83** | 1.3 | 1.4 |
| Valinomycin | 6.0E-06 | 4 | **8.19** | 0.74 | **17.44** | 4.9 | 0.5 |
| Verapimil | 1.5E-05 | 4 | **-1.62** | 0.41 | **2.03** | 1.09 | -0.8 |
| Veratridine | 9.9E-06 | 4 | **0.75** | 1.56 | **0.84** | 0.81 | 0.9 |
| Vinblastine | 7.3E-06 | 4 | **40.81** | 4.26 | **56.89** | 7.05 | 0.7 |
| Vinpocetine | 1.9E-05 | 4 | **13.72** | 1.73 | **2.14** | 0.28 | 6.4 |
| W7 | 1.8E-05 | 4 | **15.99** | 1.54 | **25.33** | 4.53 | 0.6 |
| WIN 55,212-2 mesylate | 1.3E-06 | 4 | **1.91** | 0.42 | **1.16** | 0.12 | 1.7 |
| Wiskostatin | 1.6E-05 | 4 | **131.4** | 6.66 | **76.09** | 19.4 | 1.7 |
| Wortmannin | 1.6E-05 | 3 | **100.29** | 2.96 | **113.67** | 1.51 | 0.9 |
| WY-14643 | 1.3E-06 | 4 | **2.13** | 2 | **0.32** | 0.71 | 6.6 |
| Y-27632 | 2.7E-05 | 4 | **17.51** | 11.4 | **7.81** | 9.77 | 2.2 |
| YC-1 | 2.2E-05 | 4 | **73.03** | 3.81 | **70.65** | 6.5 | 1.0 |
| Yohimbine | 1.7E-05 | 4 | **8.31** | 0.72 | **0.91** | 1.7 | 9.1 |
| YS035 | 1.9E-05 | 4 | **-0.28** | 0.25 | **-0.26** | 0.54 | 1.1 |
| Zaprinast | 2.5E-05 | 4 | **-3.06** | 0.22 | **-0.67** | 0.17 | 4.6 |
| Zardaverine | 2.5E-05 | 4 | **0.05** | 1.19 | **0.44** | 0.26 | 0.1 |
| ZM226600 | 1.8E-05 | 4 | **6.68** | 1.9 | **-0.22** | 1.02 | -30.6 |
| ZM336372 | 1.7E-05 | 4 | **23.01** | 1.31 | **4.06** | 1.85 | 5.7 |
| Z-prolyl-prolinal | 2.0E-05 | 4 | **1.24** | 1.75 | **-0.05** | 1.11 | -25.1 |
| Z-VAD(OMe)-FMK | 1.4E-05 | 4 | **16.91** | 1.37 | **21.37** | 3.13 | 0.8 |

**Supplementary Table 2: EC50 generation of hypoxia specific hits in T47D spheroids under hypoxic conditions.** Dead cells were stained with SytoxGreen and fluorescent intensities in spheroids were measured. Values were normalized with solvent (DMSO) control (0%) and Staurosporine (10 µM) as positive control (100%) for cell death. EC50 values were determined in ≥3 independent experiments. * phenothiazine hit expansion.

| **Compound** | **EC50 (cell death) in T47D tumor spheroids [M]** |
| --- | --- |
|  |  |
| **Trifluoperazine** | 8.18E-06 |
| ***Fluphenazine** | 3.83E-06 |
| **ML9** | 3.86E-06 |
| ***Chlorpromazine** | 4.54E-06 |
| **ML7** | 9.29E-06 |
| ***Thioridazine** | 3.03E-06 |
| **Tamoxifen** | inactive |
| **Cytochalasin B** | 3.30E-06 |
| **E6 berbamine** | 4.12E-06 |
| **Wiskostatin** | NA |
| **Latrunculin B** | inactive |
| **GF-109203X** | 9.62E-06 |

**Supplementary Table 3: Chemical structure of hypoxia specific hits**red=basic center

| **Compound** | **Chemical Structure** |
| --- | --- |
| **Trifluoperazine** | 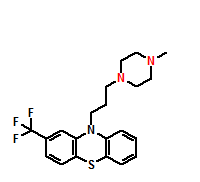 |
| **Fluphenazine** | 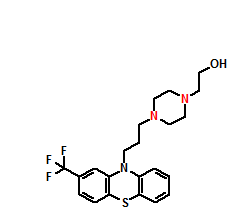 |
| **ML9** | 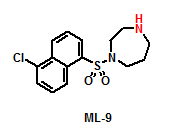 |
| **Chlorpromazine** | 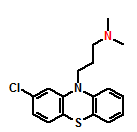 |
| **ML7** | 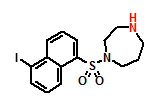 |
| **Thioridazine** | 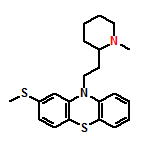 |
| **Tamoxifen** | 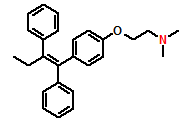 |

**Supplementary Table 4: Analysis of all protein-encoding genes by deep sequencing of HCT116 cells treated for 24 h with either 1 mM DFO or 1mM DFO + 5 µM Fluphenazine identifies several HIF-1 target genes to be upregulated (≥1,3** **fold increased expression and adjusted p-value <0.05) upon cotreatment.**

| **Gene** | **Fold Change** | **p value** |
| --- | --- | --- |
|  |  |  |
| *ADM* | **1.6** | 1.9E-05 |
| *ANGPT2* | **3.7** | 1.6E-05 |
| *BNIP3* | **1.3** | 3.7E-05 |
| *CTGF* | **2.3** | 7.1E-09 |
| *DDIT4 (REDD1)* | **2.1** | 3.8E-07 |
| *EGLN3* | **1.7** | 2.4E-07 |
| *MCL1* | **1.4** | 1.2E-06 |
| *NDRG1* | **1.3** | 1.4E-06 |
| *NR4A1* | **1.5** | 3.6E-05 |
| *PFKFB3* | **1.6** | 3.3E-07 |
| *PLAUR (uPAR)* | **1.5** | 5.4E-06 |
| *SERPINE1* | **1.7** | 5.4E-07 |
| *SLC2A3* | **1.8** | 2.5E-09 |
| *SNAI1* | **1.7** | 2.0E-05 |
| *SNAI2* | **2.6** | 3.6E-06 |
| *STC2* | **2.2** | 2.4E-08 |
| *VEGF* | **2.1** | 5.6E-09 |
| *ZEB1* | **2.0** | 2.8E-06 |
