# Supplementary figures and images for: Functional inhibition of acid sphingomyelinase by Fluphenazine triggers hypoxia-specific tumor cell death
Source: Cell Death Dis. 2017 Mar 30;8(3):e2709–. doi: 10.1038/cddis.2017.130 (PMC5386533; doi:10.1038/cddis.2017.130)

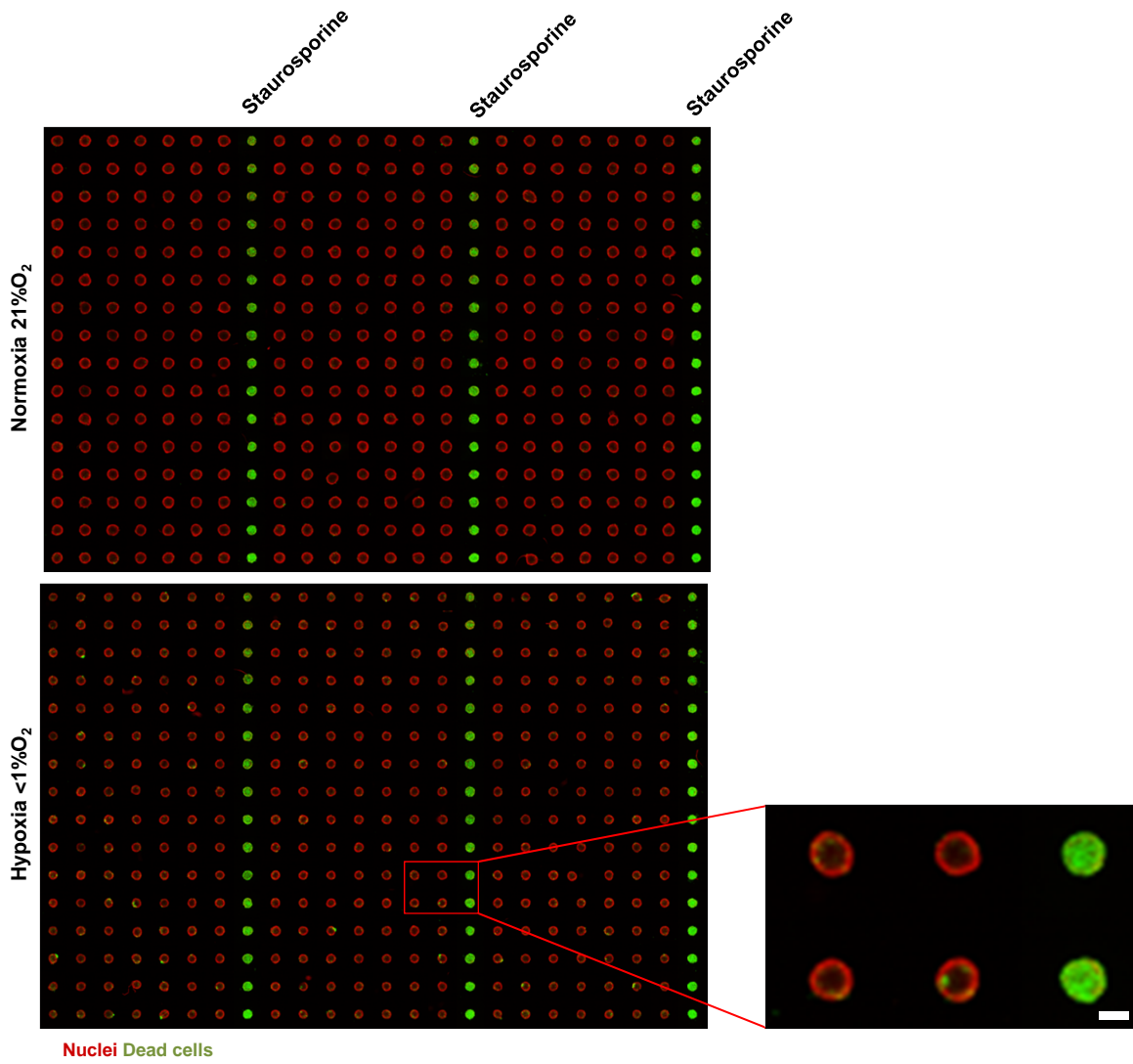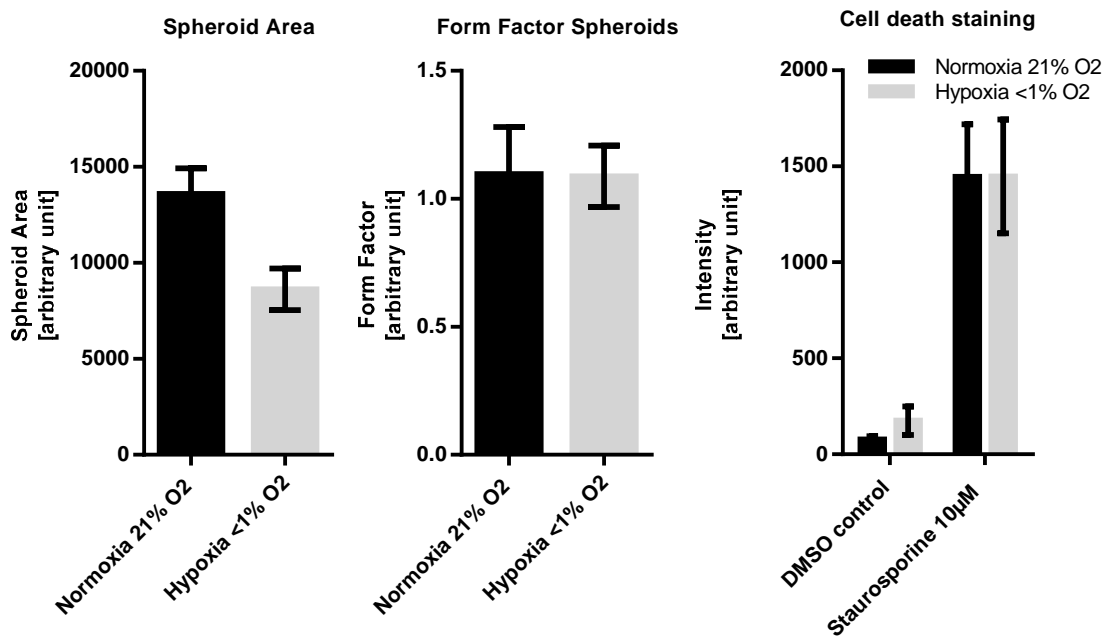

Supplement: Supplementary Figure S1 [file cddis2017130x2.pdf]

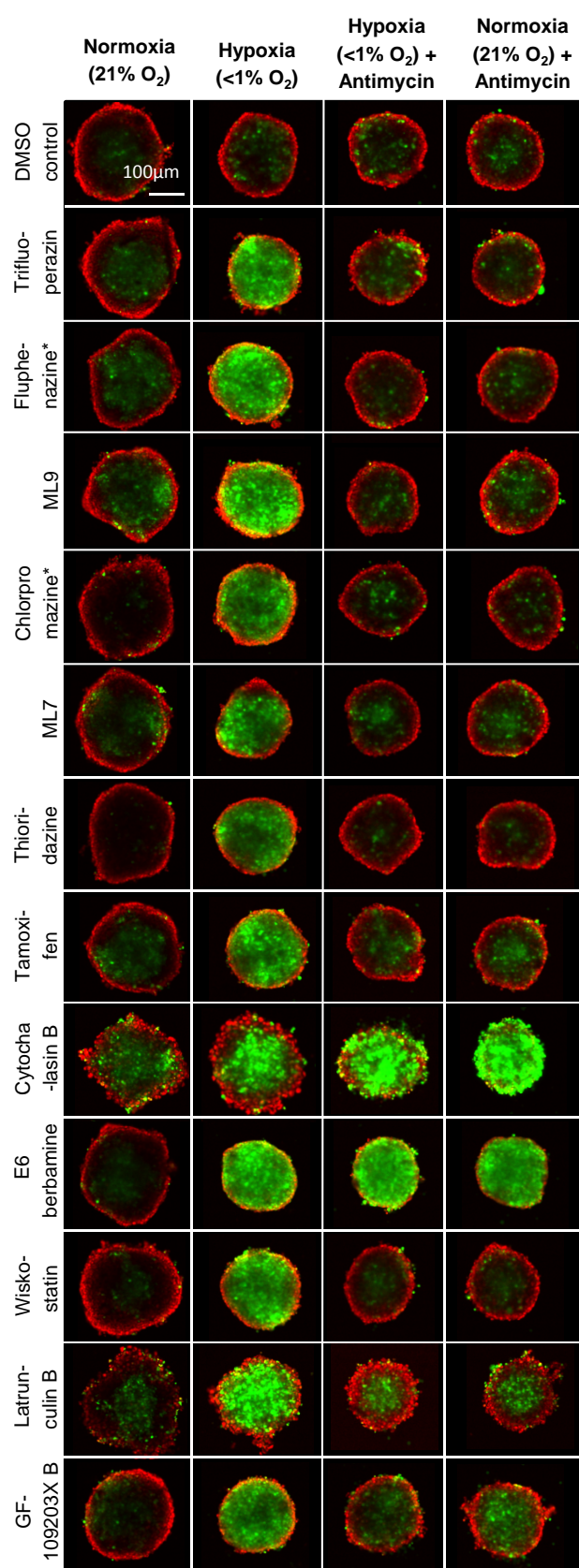

Nuclei Dead cells

Supplement: Supplementary Figure S2 [file cddis2017130x3.pdf]

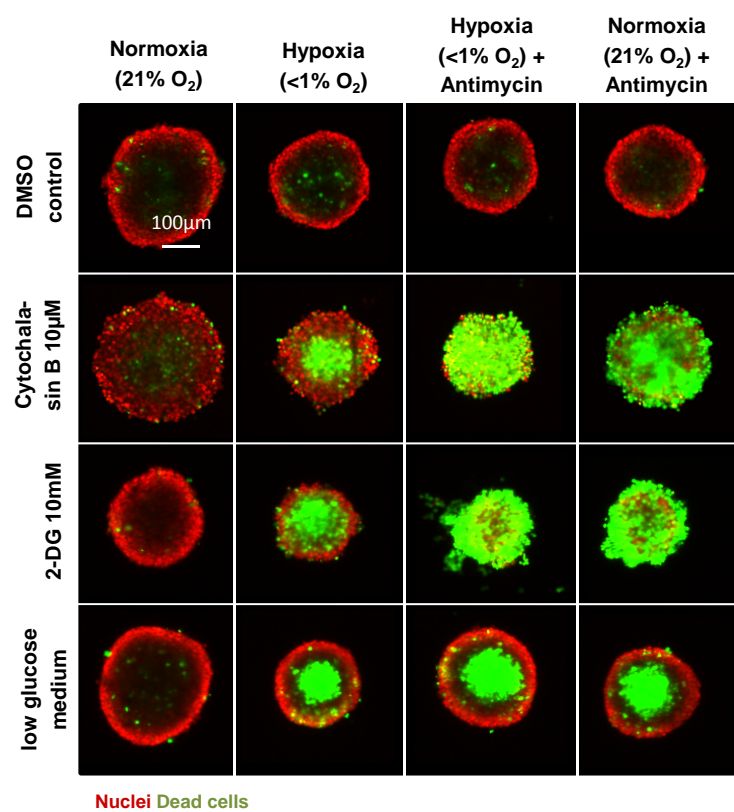

Supplement: Supplementary Figure S3 [file cddis2017130x4.pdf]

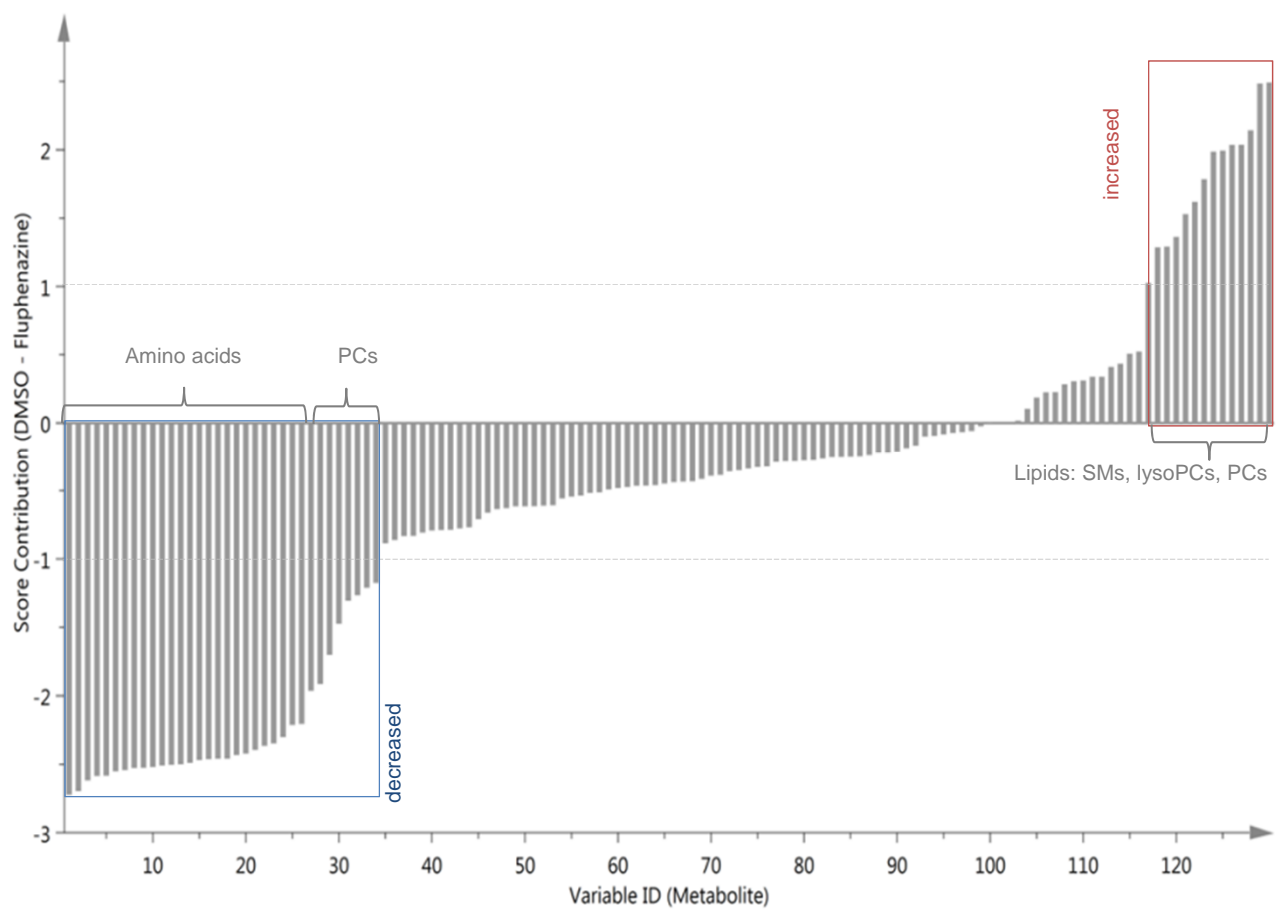

Supplement: Supplementary Figure S4 [file cddis2017130x5.pdf]

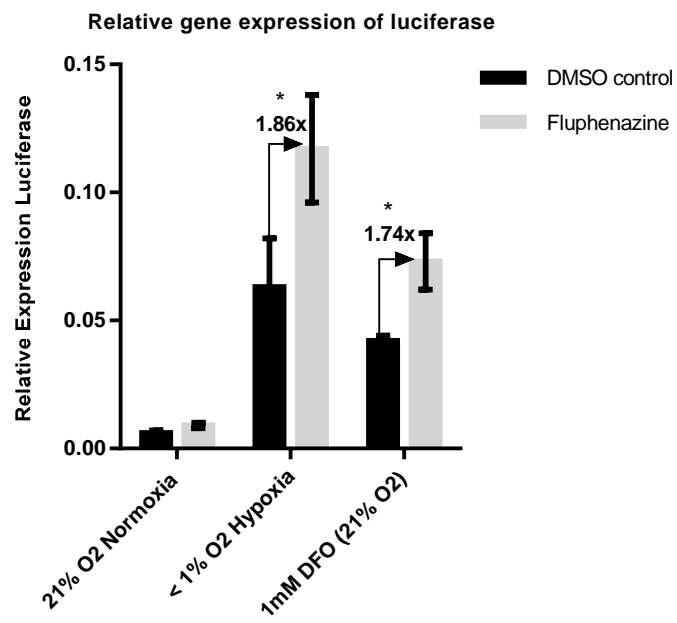

Supplement: Supplementary Figure S5 [file cddis2017130x6.pdf]

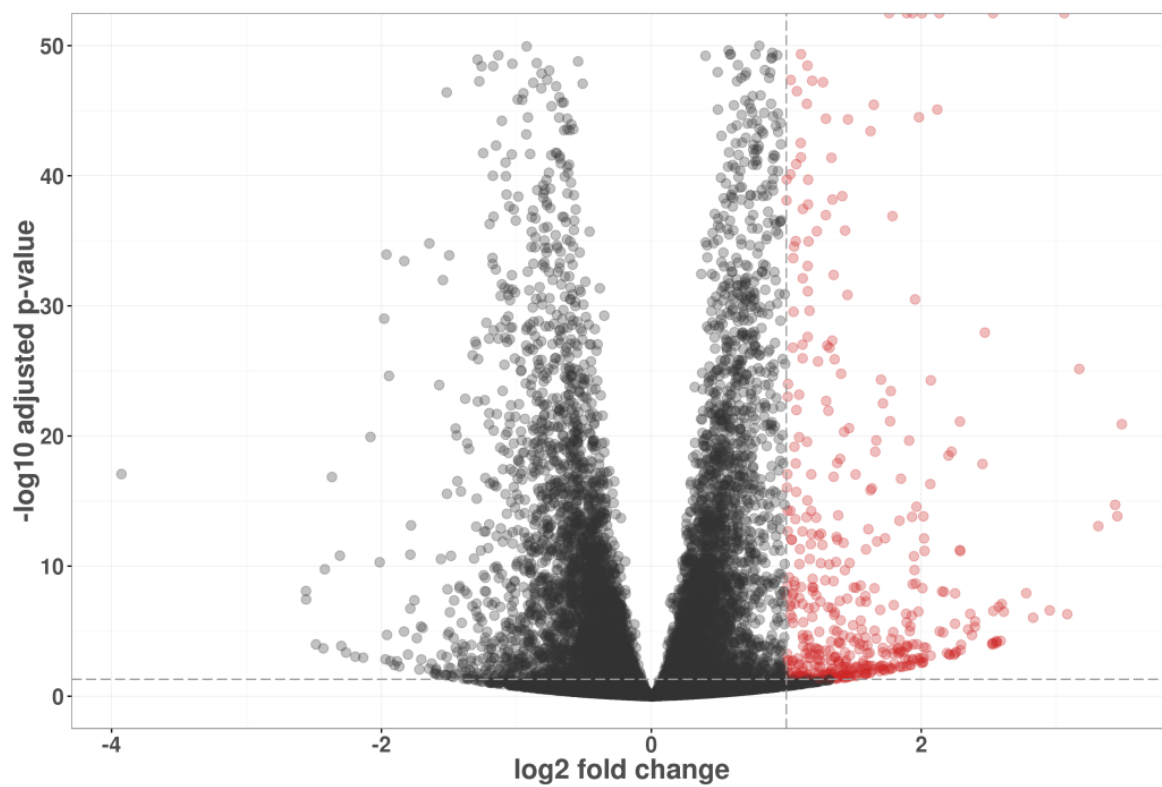

Supplement: Supplementary Figure S6 [file cddis2017130x7.pdf]

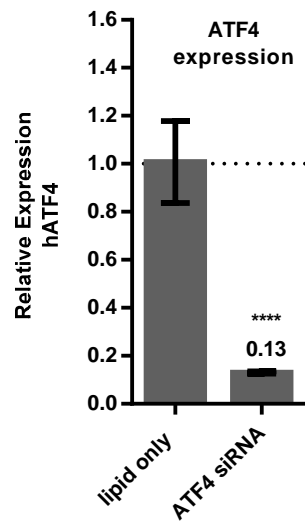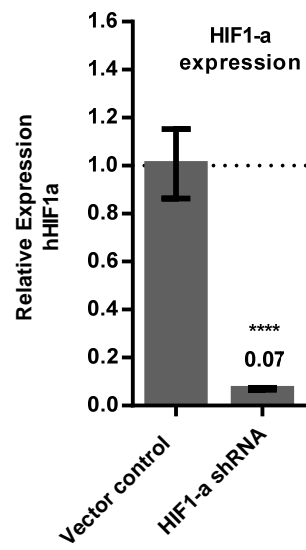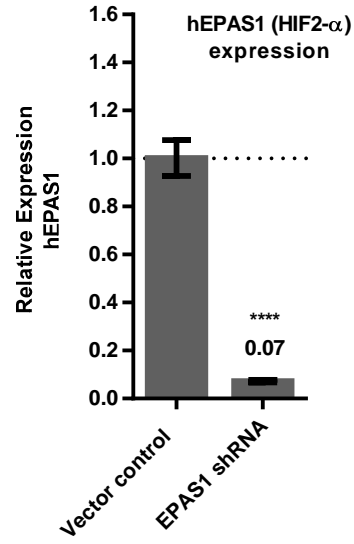

Supplement: Supplementary Figure S7 [file cddis2017130x8.pdf]
