# Supplementary material for: Functional inhibition of acid sphingomyelinase by Fluphenazine triggers hypoxia-specific tumor cell death
Source: Cell Death Dis. 2017 Mar 30;8(3):e2709–. doi: 10.1038/cddis.2017.130 (PMC5386533; doi:10.1038/cddis.2017.130)
Supplement: Supplementary Figure Legends [file cddis2017130x9.docx]

**Supplementary Figure 1:** **3D spheroid generation from single cell suspension in agarose-covered 384 Well MTPs allows spheroid formation with high intra-well and intra-plate reproducibility**. Single cell suspension of HCT116 cells was seeded in agarose-covered 384 Well plates. Cells were incubated for 4 days to form spheroids, followed by 3 days incubation either under normoxia or hypoxia (treated with either DMSO control or 10 µM Staurosporine). After 7 days spheroids were stained with Hoechst (red) and dead cells were stained with SytoxGreen (green). Scale bar 500 µm. Form factor= ratio of spheroid length to its breadth (spheroid with a form factor of 1 represents a circle). Bars show mean with SD.

**Supplementary Figure 2:** **High-Content Screen and hit expansion (marked with *) on HCT116 spheroids identifies 12 compounds that induce hypoxia specific cell death**. Spheroids were grown for 4 days in normoxic conditions, followed by 3 days compound treatment (5 µM) and incubation either in normoxia, hypoxia, hypoxia + Antimycin (200 nM) or normoxia + Antimycin (200 nM). Spheroid nuclei were stained with Hoechst (red) and dead cells were stained with SytoxGreen (green). Representative images of multiple experiments shown (n≥3). Scale bar 100 µm.

**Supplementary Figure 3:** **GLUT or Glycolysis inhibition sensitizes cells in 3D spheroids to cell death in hypoxia.** HCT116 spheroids were treated with different compounds for 3 days either under normoxia, hypoxia, hypoxia + Antimycin (200 nM), normoxia + Antimycin (200 nM). For incubation with low glucose medium, spheroids were grown in normal culture medium, which was replaced after 4 days with medium without glucose. After 7 days spheroids were stained with Hoechst (red) and dead cells were stained with SytoxGreen (green). Representative images of multiple experiments shown (n≥3). Scale bar 100 µm.

**Supplementary Figure 4:** **Fluphenazine alters cellular lipid composition of HCT116 cells.** Global analysis of 188 metabolites in HCT116 cells treated for 24 h with either DMSO control or 5 µM Fluphenazine (median of 4 replicates). PC: Phosphocholine, lysoPC: Lysophosphatidylcholine, SM: Sphingomyelin.

**Supplementary Figure 5**: **Luciferase mRNA level increases upon Fluphenazine treatment in HIF stabilized conditions or hypoxia**. HCT116-HRE-Luc cells were treated for 24 h with 5 µM Fluphenazine or DMSO and incubated either under normoxia, hypoxia or in combination with DFO. Cell pellets were collected and mRNA extracted. Gene expression analysis was performed for luciferase by RT-qPCR. hRP-L32 was used as reference gene to determine relative expression level. Bars show mean with SD (n=3).

**Supplementary Figure 6:** **Analysis of all protein-encoding genes (~ 20.000 genes) by deep sequencing of Fluphenazine treated HCT116 cells.** Volcano plot showing gene expression changes of cells treated with 1 mM DFO + 5 µM Fluphenazine vs 1 mM DFO alone. In red: genes upregulated at least 2-fold in co-treated cells compared to DFO alone treated cells (adjusted p-value <0.05). Mean of 4 replicates.

**Supplementary Figure 7:** **Knockdown efficacies in HCT116 spheroids of HIF1-a shRNA, EPAS1 (HIF2-a) shRNA and ATF4 siRNA.** siRNA treated cells grown as spheroids (for ATF4) or spheroids from HCT116 cells stably transfected with HIF shRNA (see material and methods) were harvested and mRNA extracted. Ct values of each sample were normalized with the internal control RPL32 and normalized to the lipid only or vector control sample. Bars show mean with SD (n=3).
